# Asymmetric Donor–Acceptor 2,7-Disubstituted Fluorenes and Their 9-Diazoderivatives: Synthesis, Optical Spectra and Photolysis

**DOI:** 10.3390/molecules30020321

**Published:** 2025-01-15

**Authors:** Andrei I. Savchenko, Vladimir N. Belov, Mariano L. Bossi, Stefan W. Hell

**Affiliations:** 1 Department of NanoBiophotonics, Max Planck Institute for Multidisciplinary Sciences (MPI-NAT), Am Fassberg 11, 37077 Göttingen, Germany; andrei.savchenko@mpinat.mpg.de (A.I.S.);; 2 Department of Optical Nanoscopy, Max Planck Institute for Medical Research (MPI-MR), Jahnstraße 29, 69120 Heidelberg, Germany; mariano.bossi@mr.mpg.de

**Keywords:** fluorescence, photoactivation, organic synthesis, diazo compounds, fluorenes

## Abstract

In a search for dyes photoactivatable with visible light, fluorenes with substituents at positions 2 and 7 were prepared, and their absorption and emission spectra were studied. In particular, the synthesis route to 9-diazofluorenes with 2-(N,N-dialkylamino) and N-modified 7-(4-pyridyl) substituents was established. These compounds are initially non-fluorescent, undergo photolysis with UV or blue light, and—in non-polar media—provide orange- to red-emitting products with a large separation between absorption and emission bands. Irradiation of non-fluorescent 9-diazoderivative **20** in dioxane with the light of 365 nm or 470 nm was accompanied by strong fluorescence gain (10 to 20 times), orange–red emission, and a large Stokes shift of photoproducts, which structurally relate to fluorescent betaine **13** (model compound without diazo group). Photolysis of **20** in protic solvents (ROH = MeOH, H_2_O) provided clean transformation to C^9^-OR derivatives, though the emission gain in protic solvents was low.

## 1. Introduction

**Figure 1:**
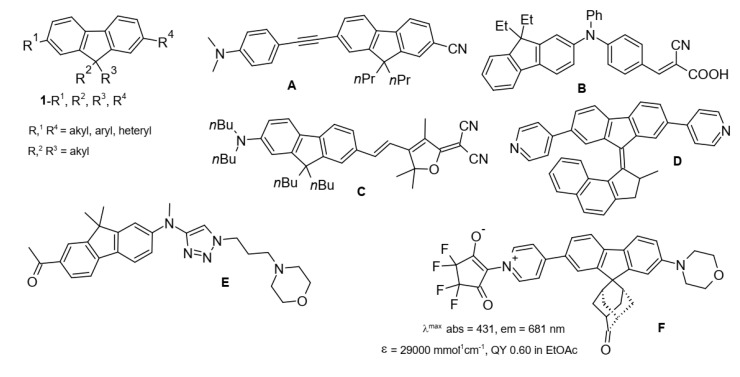
9,9-Disubstituted fluorenes for various applications (see text for details).

Fluorenes and their 9,9-dialkyl derivatives **1**-R^1^,R^2^,R^3^,R^4^ are well-known photoluminescence dyes used in various applications [1]. The recent examples include compounds for OLED devices (e.g., **A**) [2], solar cells (**B**) [3], nonlinear optical materials (**C**) [4], molecular motors (**D**) [5], and two-photon bioimaging (**E**) [6]. (Figure 1).

For varying the photophysical properties, the donor–acceptor substituents were introduced into fluorenes **A**, **B**, and **E** [2,3,6]. For 9,9-dimethylfluorenes, the influence of substituents in positions 2 and 7 on photophysical properties is well documented [7]. 9,9-Dialkylfluorenes themselves can serve as electron donors when incorporated into larger fluorophores [3].

The nature of substituents at positions 2 and 7 of the fluorene core influences the emission spectra most significantly [7]. The presence of aryl substituents induces and enhances the emission and shifts the fluorescence emission maxima within the broad spectral region. For example, compound **F** (Figure 1), possessing a 4-pyridyl betaine fragment, showed the absorption and emission maxima at 431 nm and 681 nm, respectively (EtOAc) [8].

9-Diazofluorene and 2-substituted 9-diazofluorenes were reported to be non-fluorescent [9]. Their irradiation with UV light causes the extrusion of nitrogen and the formation of a carbene followed by its reaction(s) with the solvent (i.e., by insertion into O-H or C-H bonds) [10,11]. These insertion products are typically fluorescent if the solvent does not reduce the emission efficiency. (Emission tends to be high in apolar solvents but decreases in polar environments). This phenomenon allowed, for example, depth-dependent photolabeling of a cell membrane as a hydrophobic domain [9].

**Scheme 1:**
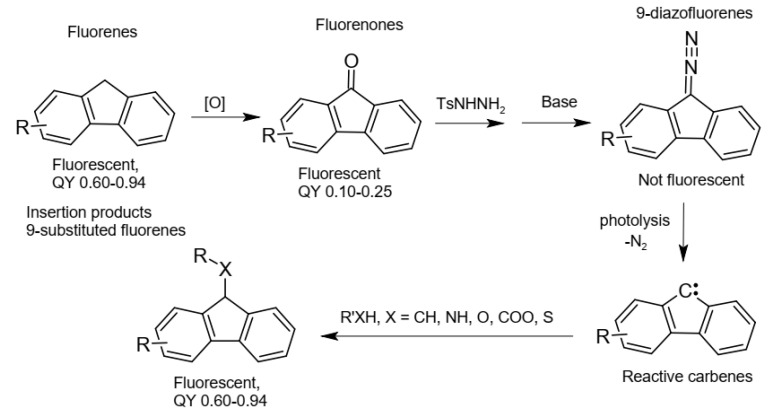
9,9-Diazofluorenes: preparation, photolysis, and carbene insertion [9,10,11].

The photoconversion of a “dark” (non-fluorescent) 9-diazofluorene to “bright” (fluorescent) products formed via carbene insertion can occur by irradiation with 365–470 nm light [9,10]. These observations suggest that 9-diazofluorenes can be considered as irreversibly photoconvertible compounds (Scheme 1). As mentioned above, their spectral and photophysical properties may be influenced by substituents at C-2 and C-7 [7]. In particular, we were interested in compounds that can be photoactivated and become fluorescent upon irradiation with visible light (>400 nm). This property is rare, as the majority of “caged” (masked) fluorophores are well activatable only with UV light. Therefore, the goal of the present work was to find and combine appropriate substituents attached to C-2 and C-7 of the 9-diazofluorene core (these positions allow direct conjugation), which can provide dyes (1) with strong absorption in the visible range, (2) undergoing photolysis (elimination of N_2_) with visible light, and (3) giving products emitting possibly in the orange or red spectral region. In particular, the synthesis route to such dyes had to be established.

## 2. Results and Discussion

### 2.1. Synthesis and Spectra

**Scheme 2:**
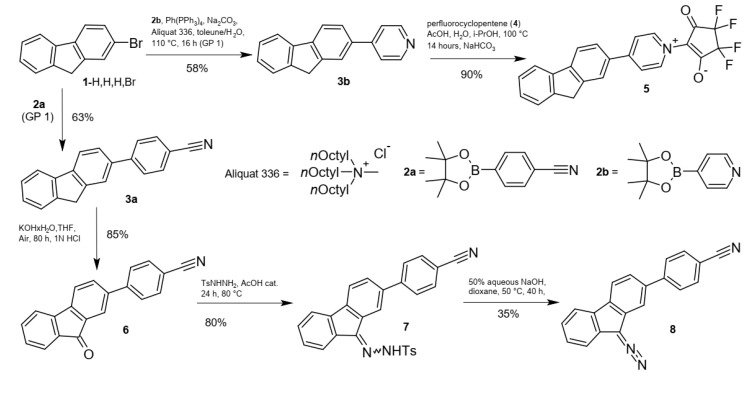
Preparation of model compound **5** and 9-diazofluorene **8**.

The model compounds in Scheme 2 were prepared from 2-bromofluorene (**1**-H,H,H,Br) by using the Suzuki reaction [12] in the presence of Aliquat 336 and Pd(PPh_3_)_4_. In particular, fluorenes **3a** [13] and **3b** [14] (Scheme 2) were prepared in 63% and 58% yields by coupling bromide **1**-H,H,H,Br with pinacol esters **2a** and **2b**. The pyridyl compound **3b** in the reaction with octafluorocyclopentene (**4**) [15] was converted to betaine **5** in 90% yield. Comparison of the optical properties of **3b** and **5** revealed a 120 nm red shift in the photoluminescence of betaine **5** and confirmed the validity of this derivatization approach.

Compound **3a** was oxidized in 85% yield to fluorenone **6** [16]. Fluorenone **6** was transformed first into tosylhydrazone **7** (by applying the known procedure [5] in ethanol and using acetic acid as a catalyst), and then compound **7** was converted to diazoderivative **8** in 26% overall yield [5].

**Figure 2:**
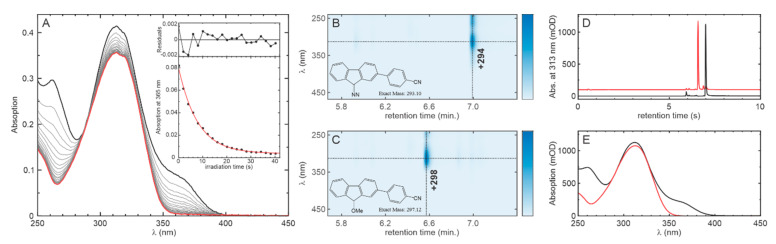
Photolysis of 9-diazofluorene **8** (10 µM) in MeOH under irradiation with 365 nm light. (**A**) Absorption changes upon irradiation and the transient at 365 nm (inset); the first and the last spectra are plotted with black and red lines, respectively, and the intermediate spectra with gray lines. A fit to a monoexponential function (red line) and the corresponding residuals are also presented. Chromatograms of the starting solution (**B**) and the solution obtained after photolysis (**C**) zoomed in on the area of interest. (**D**) Signal at the absorption maxima (313 nm) for the complete chromatograms, for the starting compound (black line), and the photolyzed solution (red line). (**E**) Absorption spectra of the main peaks, at retention times of 7.0 min (black line) and 6.5 min (red line), respectively. The curves in (**D**,**E**) correspond to the horizontal and vertical lines in (**B**,**C**), respectively.

The photolysis of compound **8** was studied under irradiation with 365 nm light in diluted methanol solution (Figure 2). A smooth transformation of the starting diazocompound **8** to a product with a shorter retention time in HPLC on reversed phase (C_18_) was observed (Figure 2B–D). The molecular mass of the product corresponded to the expected **8-OMe** derivative, and its absorption (red line in Figure 2E) resembles the absorption at the end of the photolysis (Figure 2A, red line), where the differences can be ascribed to solvent.

Comparison of the absorption spectra of the starting material and the photoproduct **8-OMe** in Figure 2A,E (black and red lines, respectively) shows that the shoulder at ca. 370 nm in compound **8** (characteristic for 9-diazofluorenes) disappeared. The clean reaction confirmed that 9-diazofluorenes are indeed good candidates for photoconvertible dyes. Therefore, we prepared further model compounds in order to study structure-spectra relationships in more detail.

**Scheme 3:**
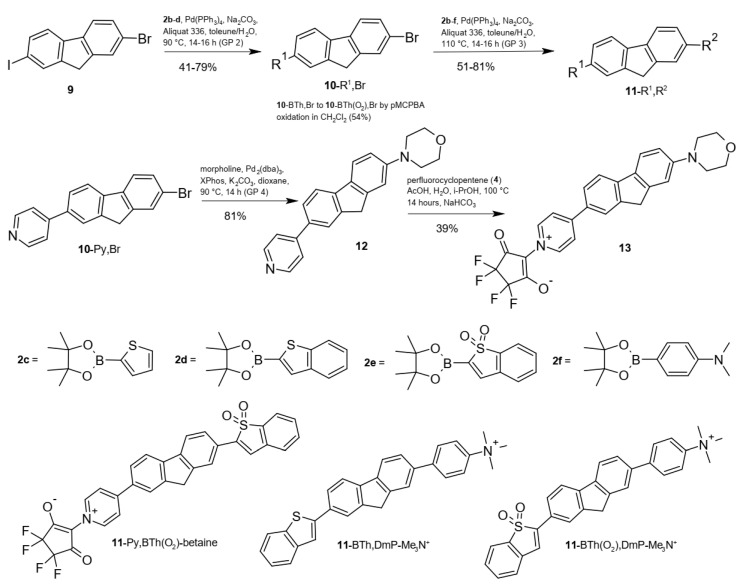
Preparation of 2,7-disubstituted fluorenes **10**-Br,R^1^, **11**-R^1^,R^2^, **12** and model betaine **13**. For substituents R^1^ and R^2^, see Table 1.

In particular, thiophenyl-(Th), benzthiophenyl-(BTh), S,S-dioxo-1-benzothiophen-2-yl-[BTh(O_2_)], and 4-(N,N-dimethylamino)phenyl-(DmP) fragments were introduced into position 7 of compound **3b**. The product structures are given in Table 1 after Scheme 3.

The synthesis was performed starting from 2-iodo-7-bromofluorene (**9**). The chemo-selective Suzuki coupling involving boronic esters **2b–d,f** was performed at 90 °C by applying the general procedure (GP2) [12] to prepare 2-bromo-7-substituted fluorenes **10**-Br,R^1^ by substitution of the iodine atom. Compound **10**-Br-BTh(O_2_) was prepared by oxidation of compound **10**-Br-BTh with MCPBA in CH_2_Cl_2_ [17].

The second Suzuki coupling of 2-bromo-fuorenes **10**-Br-R^1^ with boronic esters **2b** (4-Pyridyl-) and **2f** (4-Me_2_NPh-) was performed at 110 °C using a similar protocol (GP3) [12] and led to 2,7-disubstituted fluorenes **11**-R^1^,R^2^. Compound **11**-Py,Py was isolated as a symmetric side product with 4-pyridyl groups replacing both halogen atoms in the first Suzuki coupling step. Compound **11**-Py,DmP (Table 1) was prepared starting from bromide **10**-DmP,Br, which, in turn, was obtained according to GP2 in 70% yield. Compound **12** was synthesized in 81% yield from morpholine and fluorene **10**-Br,Py in dioxane [18]. Betaine **13** was obtained from compound **12** under conditions [15] specified in Scheme 3. Photophysical properties of prepared fluorenes are given in Table 2.

The data in Table 2 show that most of the compounds have UV, violet, or blue emission, which is not optimal for fluorescence microscopy (due to background and autofluorescence emission of the cellular structures or tissues). However, we observed important trends, which helped to propose structures of green- to red-emitting fluorenes. For example, the spectral properties of **11**-Th,Py and **11**-BTh,Py having thiophene or benzothiophene substituents were quite similar. Compound **10**-BTh(O_2_),Br was converted to compound **11**-Py,BTh(O_2_)-betaine [via intermediate **11**-Py,BTh(O_2_)]. The positions of emission bands did not change much upon this transformation. The optical properties of **11**-Py,BTh(O_2_) were not measured due to low solubility in dioxane. Thus, the use of thiophene or benzothiophene residues as donor groups turned out to be insufficient for providing the red emission required for applications in live or material science.

**Scheme 4:**
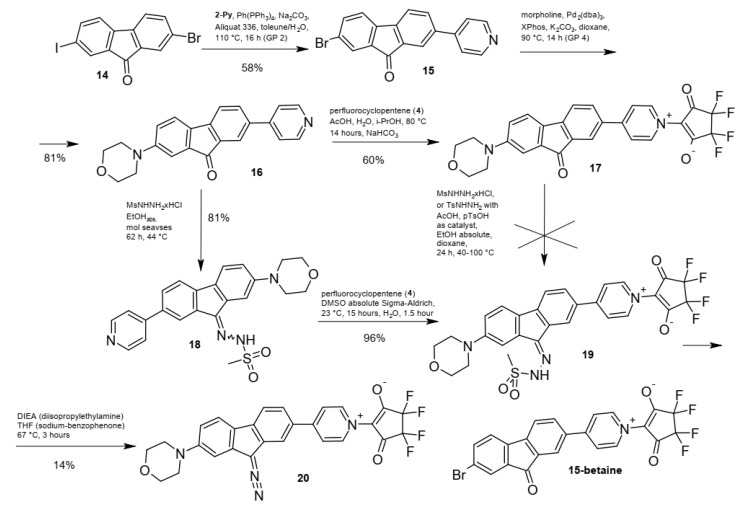
Synthesis of diazo compound **20**: elaboration of the sequence of steps and optimization.

The presence of a secondary amine at C-2 combined with a 4-pyridyl fragment at C-7 provided a 25 nm bathochromic shift of absorption and an 81 nm shift of the emission band in compound **12**, compared with the starting compound **10**-Py,Br. The introduction of the betaine group by reaction of the pyridine moiety in compound **12** with octafluorocyclopentene (**4**) produced compound **13** with large 94 nm and 178 nm bathochromic shifts in absorption and emission bands, respectively, relative to compound **12**. It was clear from the photolysis of model compound **8** that the products of the reaction formed via carbene intermediate did not display any substantial shift in their absorption spectrum (Figure 2). Therefore, it was expected that compound **13** (betaine) converted to 9-diazo derivative **20** (Scheme 4) would restore its optical properties upon photolysis, followed by a reaction of an intermediate carbene with solvent (insertion of carbene into O-H, N-H, or C-H bonds).

The required diazocompound **20** (Scheme 4) was prepared from 2-iodo-7-bromofluorenone **14** via 2-bromo-7-(4-pyridyl)fluorenone **15**, which was coupled with morpholine to give 2,7-disubstituted fluorenone **16**. Compound **15** was also transformed to derivative **15**-betaine to collect photophysical properties. Betaine **17** was prepared from fluorenone **16** in 60% yield by the standard procedure mentioned above for compound **5** [15] (Scheme 2). All attempts to prepare tosylhydrazone **19** from ketone **17** (both contained betaine fragments) resulted in a contaminated product, even when we tried to optimize the reaction conditions. For example, we used hydrochloride of methanesulfonyl hydrazide [19] and prepared hydrazone **19** from compound **17** by carrying out the reaction in absolute ethanol. However, due to the hydrolysis of **19** to **17** with water formed in the course of the reaction, the transformation of **17** to **19** was incomplete.

By changing the reaction steps (betaine and hydrazone formation), first, mesylhydrazone **18** was prepared from compound **16** in absolute ethanol in the presence of molecular sieves (81% yield). Finally, betaine **19** was synthesized from hydrazone **18** in dry DMSO at room temperature in 96% yield. The cleavage of the mesyl group could not be accomplished by using 50% aq. NaOH due to competing hydrolysis of hydrazone **19** to compound **17**. Eventually, diisopropyl ethyl amine (DIEA) in dry THF [20] was used to provide diazo compound **20** from hydrazone **19** in 14% yield after flash chromatography on silica gel (Scheme 4).

### 2.2. Photolysis of the Optimized Probe

**Figure 3:**
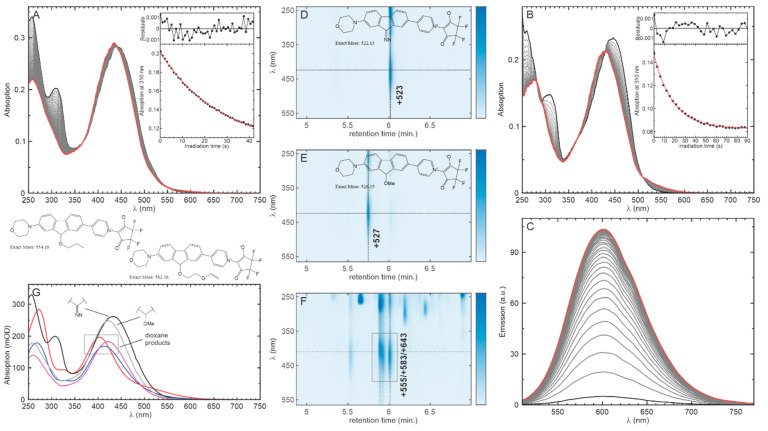
Photolysis of 9-diazofluorene **20** (10 µM) in MeOH and dioxane under irradiation with 365 nm light. Absorption changes upon irradiation in MeOH (**A**) and in dioxane (**B**), with the first and the last spectra plotted with black and red lines, respectively, and the intermediate spectra with grey lines. The insets show the transients at 310 nm, a fit to a monoexponential function (red lines), and the corresponding residuals are also presented. (**C**) Emission changes upon irradiation in dioxane (excitation at 465 nm). Chromatograms of the starting solution (**D**) and the solution obtained after photolysis in MeOH (**E**) and in dioxane (**F**) zoomed to the area of interest. (**G**) Absorption spectra of the starting compound (black line, retention time 6.0 min in (**B**)), the product of photolysis in MeOH (red line, retention time 5.7 min in (**C**)), and the main peaks of the photolysis mixture in Dioxane (retention times 5.9, 5.95, and 6.0 min in (**F**)); all spectra correspond to the vertical dotted lines in D-F, and the found *m*/*z* peak values are indicated. Two structures of the possible products obtained in dioxane are presented.

The photolysis of compound **20** was studied under irradiation with 365 nm light in diluted methanol solution (Figure 3). A clean transformation to the product of O-H insertion with the solvent, **20-OMe,** was observed (Figure 3A) and confirmed by the molecular mass of the obtained product (Figure 3E). The resulting product obtained is non or purely emissive in MeOH. Thus, photolysis was performed in identical conditions in dioxane (Figure 3B). In this case, a 20-fold increase in emission (λ_Ex_ = 465 nm) at 600 nm was observed (Figure 3C). However, a mixture of products was obtained (Figure 3F), presumably resulting from the C-O insertion of the carbene, followed by the breaking of the six-membered ring of dioxane (solvent). The absorption spectra of these products (Figure 3G) are similar to the spectra observed for **20-OMe** (Figure 3A).

**Figure 4:**
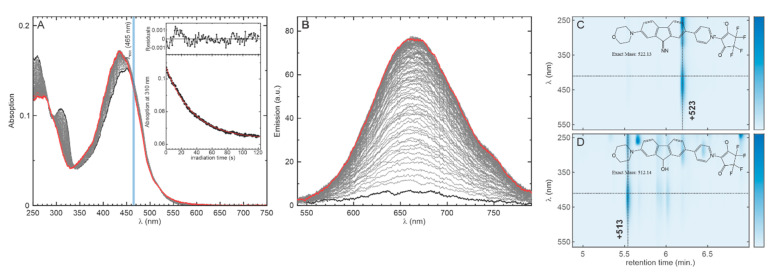
Photolysis of 9-diazofluorene **20** (10 µM) in a 10:90 (vol %) mixture of water and dioxane under irradiation with 470 nm light. (**A**) Absorption and (**B**) emission changes upon irradiation, with the first and the last spectra plotted with black and red lines, respectively, and the intermediate spectra with grey lines. The inset in A shows the transient at 310 nm, a fit to a monoexponential function (red lines), and the corresponding residuals. Chromatograms of the starting solution (**C**) and the solution obtained after photolysis (**D**), zoomed to the area of interest (the found *m*/*z* peak values are indicated).

Next, we explore the possibility of performing the photoactivation of compound **20** with visible light. To this end, a sample was irradiated in MeOH with 465–470 nm light (Figure S1) and in a 10:90 (vol %) mixture of water and dioxane (Figure 4). Under these conditions, photoactivation occurred at a somewhat lower rate than under UV irradiation (365 nm). Thus, the possibility for this compound to undergo activation with visible blue light was confirmed.

Compounds with strong push–pull substituents usually present a strong intramolecular charge transfer character in the excited state and fluorescence emission with a large Stokes shift. Stabilization of the excited state increases in polar solvents, resulting in a larger Stokes shift but lower emission efficiency; this effect is pronounced in highly polar and hydrogen donor solvents. This may explain why we did not observe emission for the product formed from compound **20** in MeOH or in aqueous buffers. To confirm this hypothesis, compound **20** was irradiated in a water/dioxane (10:90) mixture (Figure 4) with visible light (465–470 nm laser diode). We obtained a relatively clean conversion to compound **20-OH** as the main product, resulting from carbene insertion into the O-H bond of water. In addition, some by-products were obtained, probably due to a reaction with dioxane, as was observed in the previous experiment (see Figure 3). Compound **20-OH** has a slightly blue-shifted absorption with respect to compound **20-OMe** and a weak emission at 665 nm in this solvent mixture.

## 3. Materials and Methods

**Reagents and solvents** (EMSURE^®^ ACS, ISO, Reag. Ph Eur) were purchased from commercial suppliers—**Sigma-Aldrich**, (St. Louis, MO, USA), ABCR (Karlsruhe, Germany), TCI Europe, and Alfa Aesar (Thermo Scientific, Waltham, MA, USA). Anhydrous solvents were stored over molecular sieves. Deuterated solvents (C_6_D_5_N, CD_2_Cl_2_, CDCl_3_, DMSO-d_6_) were purchased from Deutero GmbH (Kastellaun, Germany). All commercially available substances were used without further purification. The reactions were performed with magnetic stirring. Oil baths were used for heating the reaction mixtures, and the bath temperatures were given as reaction temperatures. Evaporations in vacuo were performed in a rotary evaporator with bath temperature not exceeding 40 °C. For small-scale reactions, a thick-walled glass tube with a screw cap (12 mL pressure tube) was used.

**Analytical TLC (normal phase)** was performed on Merck Millipore ready-to-use aluminum sheets coated with silica gel 60 (F254). Compounds were detected by exposing TLC plates to UV light (254 or 366 nm), by staining with I_2_ deposited on silica gel, or by heating after wetting with alcoholic ceric ammonium molybdate solution.

**Flash chromatography** was performed on an automated *Isolera™ One* system. Normal phase: cartridges with 20 µm silica gel (Sfär series) from *Biotage GmbH* (Uppsala, Sweden), unless specified otherwise (e.g., reversed-phase cartridge). The eluents and gradient conditions are given for each individual run. Dry loads were prepared using 10% of the material on silica gel (40–63 µm, Merck, Rahway, NJ, USA), placing this material on the top of the cartridge and using it for purification. **Preparative reverse-phase HPLC** was performed on an Interchim PuriFlash 4250 hybrid system with a 5 mL injection loop, a 200–600 nm UV–Vis, and an ELSD detector. Preparative HPLC column Eurosphere II C18, 5 μm, 250 × 20 mm, flow rate: 20 mL/min, unless specified otherwise.

**LCMS analyses** were performed on a Phenomenex analytical column, Kinetex C18, 2.6 μm, 75 × 3 mm, with a flow rate of 0.5 mL/min, with a Thermo Fisher Scientific ISQ EM mass spectrometer (coupled to the ultimate 3000 system) using a gradient of acetonitrile 20–100% over 10 min in H_2_O (0.1% *v*/*v* HCO_2_H)/MeCN (0.1% *v*/*v* HCO_2_H) mixture.

**High-resolution mass spectrometry measurements** (EI and ESI) were recorded on a MICROTOF spectrometer (Bruker, Billerica, MA, USA) equipped with an ESI ion source (Apollo) and direct injector with LC autosampler (Agilent RR 1200, Santa Clara, CA, USA) spectrometer in the Institut für Organische und Biomolekulare Chemie, Georg-August-Universität Göttingen.

**NMR spectra** were recorded at 25 °C with spectrometers: Varian (Palo Alto, CA, USA) Agilent 400-MR at 400 MHz (^1^H), 376.4 MHz (^19^F), and 100.6 MHz (^13^C); Bruker Avance III HD 500 MHz at 500 MHz and 126 MHz (^1^H, ^13^C and 2D NMR) with the BBO Prodigy probe; and Bruker Avance III Neo 600 MHz and 151 MHz (^1^H and ^13^C, 2D NMR) with the PA TBI probe (Institut für Organische und Biomolekulare Chemie, Georg-August-Universität Göttingen). Chemical shifts (δ) are reported in ppm. All ^1^H spectra are referenced to tetramethylsilane (TMS; δ = 0 ppm) using the signals of added TMS (0.03% *v*/*v*) or the residual protons of CHCl_3_ (7.26 ppm) for CDCl_3_, DMSO-*d5* (2.50 ppm) for DMSO-*d6*, pyridine-*d4* (7.58 ppm) for pyridine-*d5*, and CDHCl_2_ (5.32 ppm) for CD_2_Cl_2_. ^13^C NMR spectra are referenced to TMS (δ = 0 ppm) using the signals of added TMS (0.03% *v*/*v*) or the solvent: CDCl_3_ (77.0 ppm), DMSO-*d6* (39.51 ppm), pyridine-*d5* (135.91 ppm), CD_2_Cl_2_ (54.00 ppm). Multiplicities of signals are described as follows: s = singlet, d = doublet, t = triplet, q = quartet, dd = double of doublets, m = multiplet or overlap of non-equivalent resonances, dm = clearly resolved doublet of overlapped signals for equivalent resonances, br = broad signal. Coupling constants (*J*) are given in Hz.

**Absorption/emission spectra, fluorescence quantum yields (absolute values), excited states lifetimes.** Absorption spectra were recorded with a double-beam UV–vis spectrophotometer (*Varian 4000*) in quartz cuvettes with a 1 cm path length. Emission spectra were recorded on a Cary Eclipse fluorescence spectrometer (*Varian*). Fluorescence quantum yields (absolute values) were obtained on a *Quantaurus-QY Absolute PL* quantum yield spectrometer C11347 (*Quantaurus QY*). Excited states’ lifetimes were measured with the *Quantaurus-Tau* device with TDC Unit M12977-01 (Hamamatsu, Shizuoka, Japan).

**Photochemistry.** Irradiation experiments were performed in a home-built setup, using 365 nm (M365L2, Thorlabs, Newton, NJ, USA) and 465 nm LEDs (M470L2, Thorlabs) as irradiation sources. To monitor the advance of the reaction, we used a single-beam absorption spectrometer with a deuterium/xenon lamp (DH-2000-BAL, Ocean Optics, Ostfildern, Germany) as an illumination source (for recording absorption spectra) and a diode array spectrometer (FLAME-SUV-VIS-ES, Ocean Optics). The intensity of the irradiation light was calibrated with a chemical actinometer (Azobenzene in MeOH). The samples were kept at 20 °C and continuously stirred with a Peltier-based temperature controller (Luma 40, Quantum Northwest, Inc., Liberty Lake, WA, USA). The absorption spectra of the samples were recorded at fixed irradiation intervals. Emission spectra were recorded at a right angle using the 465 nm LED as an excitation source (with a short pulse of ca. 200 ms). LCMS experiments (Shimadzu LCMS-2020, Kyoto, Japan) were performed with the starting solution (before irradiation) and with the solutions obtained at the end of the photolysis experiments.

### 3.1. General Procedures

#### 3.1.1. General Protocol 1 (GP1) for Preparation of 2-Substituted Fluorenes

The compounds were prepared in close analogy to the literature report [12] but using Pd(PPh_3_)_4_ instead of cataCXium A Pd G3. The mixture of 2-bromo-fluorene **1** (100 mg, 0.408 mmol, 1.0 eq), boronic acid pinacol ester **2** (0.49 mmol, 1.2 eq), Na_2_CO_3_ (151 mg, 1.42 mmol, 3.5 eq), and Pd(PPh_3_)_4_ (14 mg, 12 µmol, 0.03 eq) in a 12 mL pressure tube (flask 1) was evacuated to 1 mbar and refilled with argon, repeating the operation 3 times. The mixture of toluene (2 mL), water (1 mL), and Aliquat 336 (4.5 mg) was degassed in a separate Schlenk flask (flask 2) in a similar way and added to the reagents by means of a syringe. The tube under argon was stopped with a screw cap, and the reaction was stirred at 110 °C for 16 h. The reaction mixture was cooled, diluted with the toluene/n-hexane mixture (1/1, 4 mL), stirred, and the aqueous layer was separated (if possible). The formed precipitate was filtered off, washed with the toluene/n-hexane mixture (1/1, 2 × 3 mL), and water (2 × 3 mL) and dried to give a practically pure product.

#### 3.1.2. General Protocol 2 (GP2) for Preparation of 7-Substituted 2-Bromofluorenes

The compounds were prepared in close analogy to the literature report [12] but using Pd(PPh_3_)_4_ instead of cataCXium A Pd G3. The mixture of 2-bromo-7-iodo-fluorene **9** (371 mg, 1 mmol, 1.0 eq), boronic acid pinacol ester **2** (1.05 mmol, 1.05 eq), Na_2_CO_3_ (371 mg, 3.5 mmol, 3.5 eq), and Pd(PPh_3_)_4_ (46 mg, 0.04 mmol, 0.04 eq) in a 50 mL Schlenk flask was treated as in GP1 (flask 1). The mixture of toluene (7 mL), water (3 mL), and Aliquat 336 (15 mg) was prepared and added to flask 1, as described in GP1, and the reaction was stirred at 90 °C for 14 h. The reaction mixture was diluted with EtOAc (60 mL), shaken, and the upper layer separated and washed with brine (2 × 10 mL). The combined aqueous phases were extracted with EtOAc (30 mL). The entire organic solution was dried over Na_2_SO_4_. After filtration and solvent evaporation, the solid material was subjected to flash chromatography with a dry load. Alternatively, the reaction mixture was diluted with toluene (5 mL) and, if required, with n-hexane (5 mL). The formed precipitate was separated, washed with a toluene/n-hexane mixture (1/1 mixture, 2 × 10 mL), water (3 × 10 mL), and dried.

#### 3.1.3. General Protocol 3 (GP3) for Preparation of 2,7-Disubstituted Fluorenes

The compounds were prepared in close analogy to the literature report [12] but using Pd(PPh_3_)_4_ instead of cataCXium A Pd G3. The mixture of 2-bromo-7-aryl-fluorene **10** (0.3 mmol, 1.0 eq), pinacol boronic ester **2** (1.5 eq), Na_2_CO_3_ (111 mg, 1.05 mmol, 3.5 eq), and Pd(PPh_3_)_4_ (14 mg, 0.012 mmol, 0.04 eq) in the 12 mL pressure tube was evacuated (1 mbar residual pressure) and flashed with argon; this operation was repeated 3 times. The mixture of toluene (3 mL), water (1.4 mL), and Aliquat 336 (5 mg) was degassed and added to the reagent mixture by means of the syringe as described in GP1. The reaction mixture was flashed with argon by gas-bubbling through the solution for 5 min. The tube was stopped with a screw cap under argon, and the reaction mixture was heated at 110 °C for 14 h. The reaction mixture was diluted with toluene/n-hexane (1/1) (3 mL), shaken, and the formed solid was filtered, washed with the toluene/n-hexane mixture (1/1, 2 × 3 mL), and water (2 × 3 mL). After drying, the solid material was used without further purification or purified by flash chromatography.

#### 3.1.4. General Protocol 4 (GP4) for Preparation of 2-Amino-7-(4-Pyridyl)-Disubstituted Fluorenes [18]

The mixture of 2-bromo-7-(4-pyridyl)fluorene **10b** (69 mg, 0.22 mmol, 1.0 eq), K_2_CO_3_ (77 mg, 0.56 mmol, 2.6 eq), XPhos ligand (15 mg, 32 µmol, 0.15 eq), and Pd_2_dba_3_ (10 mg, 11 µmol, 0.05 eq) in a 12 mL pressure tube was dried at 1 mbar for 3 h. Morpholine (28 mg, 0.32 mmol, 1.5 eq) and dioxane (1.1 mL) were added, and argon was bubbled through the suspension for 10 min. The tube was sealed under argon, and the reaction mixture was stirred at 90 °C for 14 h. Then, it was diluted with n-hexane (4 mL), shaken, and the formed solid was filtered off. The residue was subjected to flash chromatography (dry load) or washed with a toluene/n-hexane mixture (1/1, 2 × 3 mL), followed by water (2 × 3 mL), and dried.

#### 3.1.5. General Protocol 5 (GP5) for Preparation of Pyridinium Betaines [15]

4-Pyridyl derivative **3b** (50 µmol, 1.0 eq), as an example, was suspended in the mixture of *i*PrOH (2.0 mL), water (200 µL), and acetic acid (50 µL) in a screw cap pressure tube (12 mL) and cooled to 0 °C. Then perfluorocyclopentene 4 (67 µL, 106 mg, 0.50 mmol, 10 eq) was added using a cold syringe, which was kept in a fridge at 4 °C for 2 h. The pressure tube was stopped, and the reaction mixture was stirred at 100 °C for 14 h. The mixture was cooled, diluted with H_2_O (5 mL) and saturated aqueous NaHCO_3_ (1 mL), well mixed, and the solid was removed (filtration or centrifugation), washed with H_2_O and freeze-dried to give a crude product. The residue was used as such or purified, as indicated below for individual compounds.

## 4. Conclusions

Searching for dyes photoactivatable with visible light, we found that a 9-diazofluorene scaffold enables the creation of non-fluorescent probes (e.g., compound **20**) undergoing photolysis in non-polar media (e.g., dioxane solution) and leading to strong fluorescence gain (10 to 20 times), orange–red emission, and a large Stokes shift. Importantly, photoactivations with UV (365 nm) or visible light (465 nm) were equally successful. The use of focusable light of 465–470 nm makes these probes applicable in fluorescence microscopy with common aberration-corrected lenses. In view of the fact that photolysis of compound **20** in protic solvents (MeOH, H_2_O) provided no increase in emission intensity, 9-diazofluorenes are likely to be applicable as photoactivatable markers in material science rather than in life science.

The synthesis of diazo compounds from diaryl ketones was optimized (Scheme 4). The use of methanesulfonyl hydrazide hydrochloride (as a reagent) in alcohols in the presence of molecular sieves at moderate temperatures can be recommended, as these conditions are compatible with many functional groups in diaryl ketones. This is an important result, as diazo compounds may easily be transformed into various other functionalities (e.g., organic fluorides), which are difficult to obtain directly from a ketone. However, this methodology is working well only with diaryl ketones. Under these conditions (see above), dialkyl ketones (with all functional groups compatible with RSO_2_NHNH_2_) smoothly provide hydrazones, but further reaction with the base does not lead to the corresponding functionally substituted dialkyl diazocompounds. In this case, the classic approach based on the reaction of (functionally substituted) ketone with hydrazine followed by oxidation of hydrazone (RR’C = NNH_2_) with HgO, Ag_2_O, or other oxidants may be applied [21].

## Figures and Tables

**Table 1:** Structure and yields of 2,7-disubstituted fluorenes **10**-R^1^,Br, **11**-R^1^,R^2^.

	R^2^	Br-	4-Pyridyl-(Py)	4-Me_2_NC_6_H_4_-(DmP)
R^1^				
4-Pyridyl-(Py)		**10**-Py,Br (47)	**11**-Py,Py (37) **	**11**-Py,DmP (51)
2-Thienyl-(Th)		**10**-Th,Br (41)	**11**-Th,Py (62)	**11**-Th,DmP (38)
2-Benzothiophen-2-yl-(BTh)		**10**-BTh,Br (79)	**11**-BTh,Py (51)	**11**-BTh,DmP (92)
2-Benzothiophen-2-yl-(oxidized) [BTh(O_2_)]		**10**-BTh(O_2_),Br (54) *	**11**-BTh(O_2_),Py (81)	**11**-BTh(O_2_),DmP (90)

* compound 10-BTh(O_2_),Br was prepared by oxidation of 10-BTh,Br with MCPBA. ** Compound 11-Py,Py was isolated as a side product in preparation for 10-Py,Br.

**Table 2:** Photophysical properties—absorption and emission maxima, extinction coefficients, fluorescence quantum yields, and lifetimes of the excited states—of compounds **3a–b**, **5–6**, **8**, **10**-R^1^,Br; **11**-R^1^,R^2^; selected betaines and trimethylammonium iodides in 10 µM dioxane (MeCN) solution. For structures, see Scheme 2, Scheme 3 and Scheme 4. The excitation wavelength was $\lambda_{abs}^{max}$—10 nm.

Compound	$\lambda_{abs}^{max}$ [nm]	$\lambda_{em}^{max}$ [nm]	*ε*_max_ [M^−1^cm^−1^]	*Φ*_fl_ [%]	LT [ns]
3a (Scheme 2)	317	372	36,500	80	1.0, 10.4 *
3b (Scheme 2)	311	352	25,700	--- **	0.8
5 (Scheme 2)	390	474	34,400	80	2.7
6 (Scheme 2)	284, 318, 404sh	504	51,200, 13,500, 1000sh	25	---
8 (MeCN) (Scheme 2)	312	384	44,800	--- **	--- ***
10-Py,Br	317	354	33,900	--- **	0.2, 0.9 *
10-BTh(O_2_),Br	368	450	30,900	53	1.7
11-Py,Py	325	356, 373	50,700	--- **	0.8, 1.6 *
11-Th,Py	339	378, 394	49,000	84	0.9
10-BTh,Br	347	381, 400	57,900	84	0.8
11-BTh(O_2_),Py-betaine	402	466	21,600	72	1.7
11-Py,DmP	345	449	32,300	90	0.3, 1.4 *
11-BTh,DmP-Me_3_N^+^	350	390, 450	11,100	73	1.6
11-BTh(O_2_),DmP	400	555	33,300	84	3.5
11-BTh(O_2_),DmP-Me_3_N^+^	373	470	17,700	72	2.6
12	342	435	24,000	94	0.2, 1.6 *
13	436	613	31,700	84	3.1
15-betaine	373	508	31,000	9	2.7, 5.4 *
17 (MeCN) (Scheme 4)	407, 505	503, 741	31,700, 6800	0.5	2.2, 10.3 *
20 (MeCN) (Scheme 4)	438	500, 738	21,500	2	--- ***

* bi-exponential decay; ** the shortest possible excitation wavelength is 350 nm; therefore, for compounds with $\lambda_{abs}^{max}$ < 330 nm, measurements were technically impossible; *** non-fluorescent, decomposed upon irradiation.

## Data Availability

The data presented in this study are available in the article and Supplementary Materials.

## Supplementary Materials

The following supporting information can be downloaded at: https://www.mdpi.com/article/10.3390/molecules30020321/s1, Figure S1: Photolysis of 9-diazofluorene **20** (10 µM) in MeOH under irradiation with 470 nm light; syntheses of compounds **3a,b**, **5–8**, **10**-R^1^,Br, **11**-R^1^,R^2^, **12, 13, 15–18, 20, 15**-betaine; absorption, emission, ^1^H NMR- and ^13^C-NMR spectra of the synthesized compounds [5,13,14,17,19,20,21,22,23,24,25,26].
