# Supplementary material for: Asymmetric Donor–Acceptor 2,7-Disubstituted Fluorenes and Their 9-Diazoderivatives: Synthesis, Optical Spectra and Photolysis
Source: Molecules. 2025 Jan 15;30(2):321. doi: 10.3390/molecules30020321 (PMC11768028; doi:10.3390/molecules30020321)
Supplement: Supplementary file 1 [file molecules-30-00321-s001.zip › molecules-3396425-supplementary.pdf]

Supporting Information

Asymmetric donor-acceptor 2,7-substituted fluorenes and their 9-diazoderivatives: synthesis, optical spectra and photolysis

Andrei I. Savchenko[1], Vladimir N. Belov[1,*], Mariano L. Bossi[2], Stefan W. Hell[1,2]

[1] Department of NanoBiophotonics, Max Planck Institute for Multidisciplinary Sciences (MPI-NAT), Am Fassberg 11, 37077 Göttingen, Germany

[2] Department of Optical Nanoscopy, Max Planck Institute for Medical Research (MPI-MR), Jahnstrasse 29, 69120 Heidelberg, Germany

* Corresponding author: vladimir.belov@mpinat.mpg.de

Table of contents:

1. Photolysis of 9-diazofluorene **20** (10 µM) in MeOH under irradiation with 470 nm light

2. Synthetic procedures

4. Supplementary references

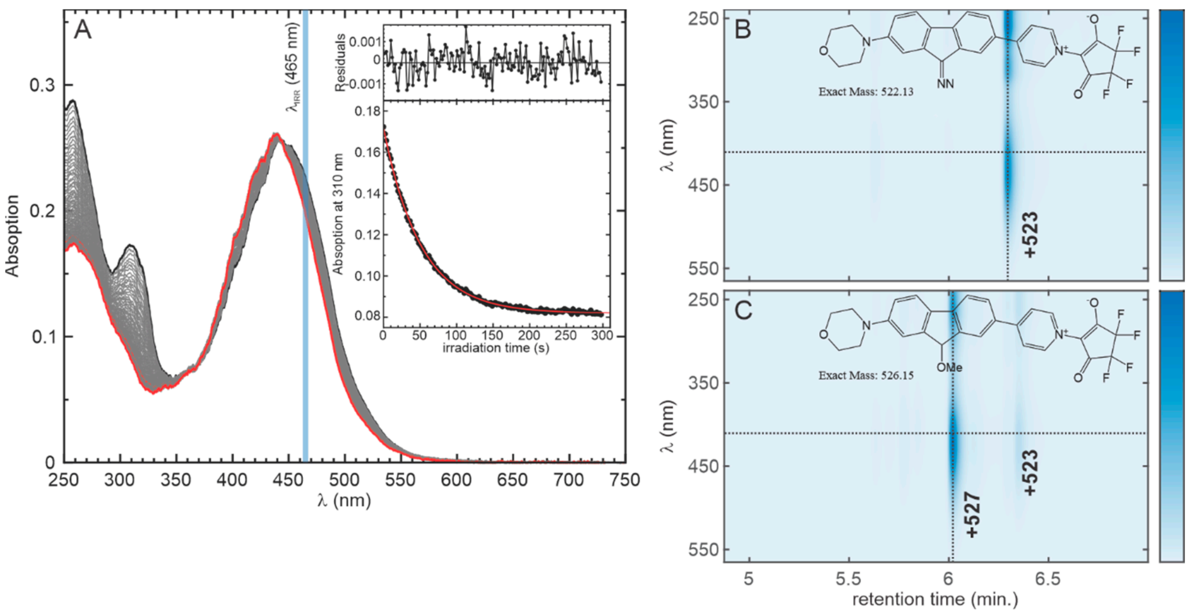

Figure S1. Photolysis of 9-diazofluorene **20** (10 µM) in MeOH under irradiation with 470 nm light. (A) Absorption changes upon irradiation, with the first and the last spectra plotted with black and red lines respectively, and the intermediate spectra with grey lines. The inset show the transient at 310 nm, a fit to a monoexponential function (red lines) and the corresponding residuals. Chromatograms of the starting solution (B) and the solution obtained after photolysis (C), zoomed to the area of interest (the

found m/z peak values are indicated). Note that there is a small amount of starting compound in the final mixture, confirmed by the mass (the difference in its retention time is due to instrumental variation).

Synthesis and Analysis

2-(4-Cyanophenyl)fluorene (3a) [13]**:**

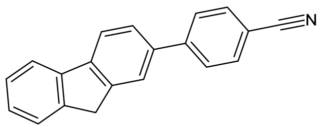

According to **GP1** and using (4-cyanophenyl)-boronic acid pinacol ester (**2a**) (112 mg, 0.49 mmol, 1.2 eq), compound **3a** was obtained as a crude product (78 mg), which was purified by flash chromatography on a 5 g cartridge. Elution with 10% EtOAc in *n*-hexane (1 CV), followed by gradient 10-100% EtOAc in *n*-hexane (10 CV), EtOAc (1 CV), 0-100% MeOH in EtOAc (5 CV), MeOH. The fraction (R_f = 0.70 in *n*-C_6H_{14}/EtOAc/MeOH = 3/1/1) was collected. After evaporation of solvents and drying, compound **3a** (69 mg, 63%) was isolated as a yellowish solid.

^{1}H NMR (400 MHz, $CDCl_3$): δ = 7.88 (d, *J*=7.8 Hz, 1H), 7.84 (br. d, *J*=7.4 Hz, 1H), 7.78 (br. dd, *J*=1.8, 0.8 Hz, 1H), 7.71-7.77 (m, 4H), 7.62 (br. dd, *J*=7.8, 1.8 Hz, 1H), 7.59 (br. d, *J*=7.4 Hz, 1H), 7.42 (br. t, *J*=7.3 Hz, 1H), 7.35 (br. td, *J*=7.3, 1.2 Hz, 1H), 3.98 (s, 2H) ppm. ^{13}C NMR (101 MHz, $CDCl_3$): δ = 145.9, 144.1, 143.5, 142.3, 140.9, 137.6, 132.6, 127.7, 127.3, 127.0, 126.1, 125.1, 123.8, 120.4, 120.2, 119.0, 110.6, 37.0 ppm.

2-(4-Pyridyl)fluorene (3b) [14]**:**

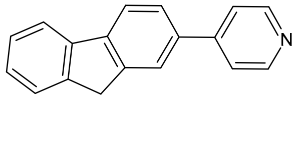

According to **GP1** and using 4-pyridylboronic acid pinacol ester **2b** (100 mg, 0.49 mmol, 1.2 eq), compound **3b** (58.4 mg, 58%) was obtained.

^{1}H NMR (400 MHz, $CDCl_3$): δ = 8.66-8.73 (m, *J* = 5.1 Hz, 2H), 7.92 (d, *J* = 8.2 Hz, 1H), 7.87 (br d, *J* = 1.6 Hz, 1H), 7.85 (br d, *J* = 8.2 Hz, 1H), 7.71-7.74 (m, 2H), 7.71 (dd, *J* = 6.3, 1.2 Hz, 1H), 7.60 (d, *J* = 7.4 Hz, 1H), 7.43 (br t, *J* = 7.0 Hz, 1H), 7.37 (dd, *J* = 8.6, 7.0 Hz, 1H), 4.01 (s, 2H) ppm. ^{13}C NMR (151 MHz, $CDCl_3$): δ = 151.0, 147.7, 144.4, 143.7, 143.6, 140.6, 135.4, 127.6, 127.1, 126.1, 125.2, 123.8, 122.1, 120.6, 120.5, 37.0 ppm. HRMS (EI) calcd for $C_{18}H_{13}N$ $[M]^+$ 243.1048, found 243.1037.

2-[4-(Fluoren-2-yl)pyridin-1-ium-1-yl]-4,4,5,5-tetrafluoro-3-oxocyclopent-1-en-1-olate (5):

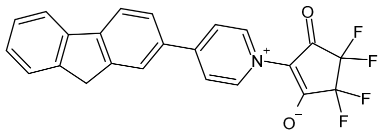

According to **GP5** and using compound **3b** (12 mg, 50 µmol), compound **5** (17.5 mg, 90%) was obtained as a yellow solid. ^{1}H NMR (400 MHz, DMSO-*d₆*): δ = 9.24-9.30 (dm, *J* = 7.2 Hz, 2H), 8.60-8.66 (dm, *J* = 7.2 Hz, 2H), 8.36-8.39 (m, 1H), 8.18 (s, 2H), 8.04-8.09 (m, 1H), 7.66-7.70 (m, 1H), 7.47 (td, *J* = 7.4, 1.2 Hz, 1H), 7.43 (td, *J* = 7.4, 1.6 Hz, 1H), 4.09 (br s, 2H) ppm. ^{13}C NMR (126 MHz, DMSO-d_6): δ = 154.0, 145.2, 144.4, 144.3, 142.0, 139.8, 131.7, 128.3, 127.4, 127.1, 125.4, 124.9, 123.4, 121.3, 121.2, 36.6 ppm; due to C-F coupling the ^{13}C signals of the betaine part were not resolved. ^{19}F NMR (376 MHz, DMSO-d_6): δ = -126.0 (s, 4F) ppm. HRMS (ESI) calcd for $C_{23}H_{13}F_4NO_2$ $[M+Na]^+$ 434.0775, found 434.0775.

2-(4-Cyanophenyl)fluorenone (6) [22]:

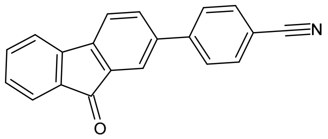

Fluorene **3a** (448 mg, 1.68 mmol, 1 eq), pellets of commercial KOH (110 mg, 1.68 mmol, 1eq) and THF (20 mL) were combined in a 100 mL round bottom flask equipped with an air condenser (30 cm) and stirred at ambient temperature for 14 h. Solid KOH (50 mg) and THF (5 mL) were added, and the

mixture stirred for additional 66 h. 1M aq. HCl (3.5 mL) was added, and the mixture was stirred for 5 min (pH 3.5). Then brine (40 mL) was added, and the formed organic layer (THF) was separated. Aqueous phase was extracted with CH_2Cl_2 (2×40 mL), and combined organic layers were dried over Na_2SO_4. The organic solution was concentrated to about 30 mL in vacuo, THF (30 mL) was added, and the entire mixture was evaporated to half. *n*-Hexane (30 mL) was added, and the formed precipitate was filtered off. The filtrate was diluted again with *n*-hexane (30 mL), and concentrated to a half to give, after cooling, an additional solid product affording in total 401 mg (85%) of compound **6** as a dark yellow solid.

^{1}H NMR (400 MHz, $CDCl_3$): δ = 7.90 (d, *J* = 1.6 Hz, 1H), 7.68-7.79 (m, 6H), 7.64 (d, *J* = 7.8 Hz, 1H), 7.58 (d, *J* = 7.4 Hz, 1H), 7.54 (td, *J* = 7.4, 0.9 Hz, 1H), 7.35 (td, *J* = 7.2, 1.2 Hz, 1H) ppm. ^{13}C NMR (101 MHz, $CDCl_3$): δ = 193.3, 144.5, 144.2, 143.8, 140.0, 135.1, 135.0, 134.4, 133.3, 132.7, 129.5, 127.4, 124.6, 122.9, 120.9, 120.7, 118.7, 111.5 ppm.

2-(4-Cyanophenyl)fluoren-9-one tosylhydrazone (7):

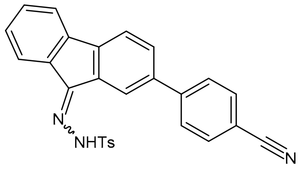

Tosylhydrazone **7** was prepared in close analogy to protocol [5]. Mixture of fluorenone **6** (380 mg, 1.35 mmol, 1.0 eq), tosylhydrazide (377 mg, 2.03 mmol, 1.5 eq) and EtOH (20 mL) were placed into a 80 mL pressure bottle, AcOH (10 drops) was added, and the reaction was stirred at 80 °C for 24 h. The mixture was diluted with water (40 mL), and the yellow suspension was filtered off; the obtained residue washed with water (2×15 mL) and dried to provide compound **7** (486 mg, 80%) as a yellow solid (a mixture of *syn*/*anti* isomers). LCMS data were obtained after the sample was treated with $NaHCO_3$ solution.

^{1}H NMR (400 MHz, DMSO-d_6), A major isomer, with ratio about 2/1: δ = 11.58 (br s, 1H), 11.49 (br s, 1H, A), 8.37 (s, 1H), 8.14 (d, *J* = 7.8 Hz, 1H, A), 7.90-8.06 (m, 12H, mixture), 7.88 (br d, *J* = 7.4 Hz, 1H),

7.81-7.85 (m, 2H, mixture), 7.63 (d, *J* = 7.4 Hz, 1H), 7.55-7.60 (m, 1H, A), 7.42-7.51 (m, 4H, mixture), 7.37 (br d, *J* = 7.4 Hz, 1H), 2.40 (m, 3H), 2.37 (s, 3H, A) ppm. HRMS (ESI) calcd for$C_{27}H_{19}N_3O_2S$ $[M+H]^+$ 450.1271, found 450.1276.

2-(4-Cyanophenyl)-9-diazofluorene (8):

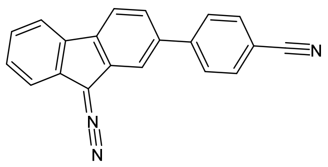

Diazocompound **8** was prepared in close analogy to the published protocol [23]. The mixture of dioxane (11 mL), tosylhydrazone **7** (234 mg, 0.52 mmol) and 50% aqueous NaOH (6 mL) was vigorously stirred under argon at 50 °C for 40 h. The reaction mixture was diluted with H_2O (6 mL) and extracted with EtOAc (3x20 mL), then the aqueous phase was diluted with brine (10 mL) and extracted by EtOAc (2x20 mL). The combined organic solutions were washed with H_2O (25 mL) and dried over $MgSO_4$. The solution was evaporated to give 250 mg of solid residue, which was purified by flash chromatography on a 5 g cartridge. Elution with gradient 0-100% CH_2Cl_2 in *n*-hexane (10 CV), CH_2Cl_2 (4 CV), gradient 0-100% MeOH in CH_2Cl_2 (5 CV). The first fraction (R_f = 0.70 in *n*-C_6H_{14}/EtOAc/MeOH = 3/1/0.1) was collected. After evaporation of solvents and drying of fractions, diazocompound **8** (53 mg, 35%) was isolated as a yellowish solid. Evaporation and drying of second fraction recovered 94 mg of starting hydrazone **7**.

^{1}H NMR (600 MHz, DMSO-d_6): δ = 8.22 (br. s, 1H), 8.21 (dd, *J*=10.0, 0.5 Hz, 1H), 8.15 (dt, *J*=7.7, 0.8 Hz, 1H), 8.04 (d, *J*=8.6 Hz, 2H), 7.95 (d, *J*=8.6 Hz, 2H), 7.76 (dt, *J*=7.8, 0.8 Hz, 1H), 7.74 (dd, *J*=8.1, 1.8 Hz, 1H), 7.44 (ddd, *J*=7.8, 7.2, 1.1 Hz, 1H), 7.36 (td, *J*=7.5, 1.0 Hz, 1H) ppm. ^{13}C NMR (126 MHz, DMSO-d_6): δ = 144.7, 136.2, 133.5, 133.1, 132.8, 130.9, 130.1, 127.7, 126.8, 124.6, 123.4, 121.7, 121.5, 119.8, 119.0, 118.5, 109.8, 63.5 ppm. HRMS (ESI) calcd for$C_{27}H_{19}N_3O_2S$ $[M+Na]^+$ 316.0845, found 316.0840. IR (KBr pellet): 2226, 2100, 2072, 1603, 1432, 820, 767, 743, 722 cm^{-1}.

2-Bromo-7-(4-pyridyl)-fluorene (10-Py,Br):

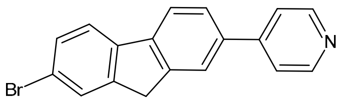

Reaction 1. According to **GP2** and using RuPhos G4 catalyst, as well as boronic acid pinacol ester **2b** (215 mg, 1.05 mmol, 1.05 eq), heating for 32 h resulted in a solid residue (381 mg) which was subjected to flash chromatography on 25 g cartridge. Elution with *n*-hexane (1CV), gradient of EtOAc 0-100% in *n*-hexane (10CV) gave the following fractions: starting 2-bromo-7-iodofluorene (**9**) (95 mg, 25%), target compound **10**-Py,Br (41 mg, 13%), bis substituted side-product **11**-Py,Py (119 mg, 37%).

Reaction 2 was performed according to **GP2** with 187 mg (0.504 mmol) of 2-iodo-7-bromofluorene (**9**) and boronic acid pinacol ester **2b** (105 mg, 0.52 mmol, 1.02 eq); at 110 °C for 17 h. Prior to the work-up, the reaction mixture was neutralised with 1.5 ml of 1 M aq. NaOH, diluted with water (15 mL) and extracted with EtOAc (2×10 mL). The combined organic solutions were dried over Na_2SO_4 and evaporated in vacuum. Flash chromatography of the solid residue (151 mg) was performed on a 10 g cartridge. Elution: 10% EtOAc in *n*-hexane (1CV), gradient 10-100% EtOAc in *n*-hexane (10CV). The following fractions were isolated: starting compound **9** (9 mg, 5%), compound **10**-Py,Br (76 mg, 47%).

^{1}H NMR (400 MHz, $CDCl_3$): δ = 8.63-8.70 (m, 2H), 7.83 (d, *J* = 7.8 Hz, 1H), 7.77-7.81 (m, *J* = 0.8 Hz, 1H), 7.68-7.72 (m, *J* = 1.2 Hz, 1H), 7.66 (d, *J* = 8.2 Hz, 1H), 7.64-7.67 (m, 1H), 7.54-7.56 (m, 2H), 7.52 (br dd, *J* = 8.2, 1.6 Hz, 1H), 3.94 (s, 2H) ppm. ^{13}C NMR (101 MHz, $CDCl_3$): δ = 150.3, 148.4, 145.5, 143.8, 141.7, 139.9, 137.0, 130.2, 128.4, 126.1, 123.6, 121.6, 121.5, 121.2, 120.5, 36.8 ppm. HRMS (EI) calcd for $C_{18}H_{12}BrN$ $[M]^+$ 321.0153, found 321.0148 and 323.0127.

2-Bromo-7-(thiophen-2-yl)-fluorene (10-Th,Br):

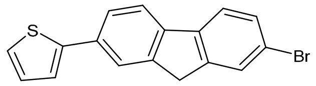

According to **GP2** and with heating at 90 °C for 3 h. The reaction mixture was diluted with EtOAc (25 mL), the aqueous layer was separated, and the organic phase dried over Na_2SO_4. Solvents were removed in vacuo to give 400 mg of crude residue, which was dissolved in CH_2Cl_2 (8 mL) and diluted

with n-C_6H_{14} for crystallisation. The formed crystalline material was filtered and washed with mixture CH_2Cl_2/n-hexane (1/2), dried at 1 mbar to afford pure compound **10**-Th,Br (136 mg, 41%).

^{1}H NMR (500 MHz, $CDCl_3$): δ = 7.77-7.79 (m, 1H), 7.74 (dd, J = 8.0, 0.5 Hz, 1H), 7.67-7.69 (m, 1H), 7.65 (ddt, J = 8.0, 1.7, 0.8 Hz, 1H), 7.63 (dd, J = 8.1, 0.5 Hz, 1H), 7.51 (ddt, J = 8.0, 1.9, 0.7 Hz, 1H), 7.36 (dd, J = 3.6, 1.2 Hz, 1H), 7.29 (dd, J = 5.1, 1.2 Hz, 1H), 7.10 (dd, J = 5.1, 3.6 Hz, 1H), 3.92 (br s, 2H) ppm. ^{13}C NMR (151 MHz, $CDCl_3$): δ = 145.3, 144.6, 143.6, 140.3, 140.1, 133.4, 130.0, 128.3, 128.1, 125.0, 124.8, 123.1, 122.5, 121.1, 120.6, 120.3, 36.7 ppm. HRMS (EI) calcd for $C_{17}H_{11}BrS$ $[M]^+$ 325.9765, found 325.9763.

2-Bromo-7-(1-benzothiophen-2-yl)-fluorene (10-BTh,Br):

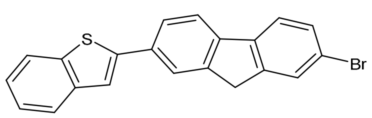

According to GP2 and using 2-bromo-7-iodo-fluorene (**9**) (1.70 g, 4.58 mmol, 1.2 eq), pinacol boronic ester **2d** (993 mg, 3.82 mmol, 1.0 eq), Na_2CO_3 (1.71 g, 16.1 mmol, 3.5 eq) and $Pd(PPh_3)_4$ (100.6 mg, 87 µmol, 0.02 eq) in a 50 mL Schlenk flask and after 3 h, a colorless precipitate was formed. The suspension was cooled, filtered, and washed with toluene/n-hexane mixture (1/1, 2×10 mL) and water (3×10 mL), and dried to afford **10**-BTh,Br (1.37 g, 79%)

^{1}H NMR (400 MHz, $CDCl_3$): δ = 7.87-7.90 (m, 1H), 7.82-7.86 (m, 1H), 7.73-7.81 (m, 3H), 7.69-7.72 (m, 1H), 7.66 (d, J = 8.2 Hz, 1H), 7.60 (d, J = 0.8 Hz, 1H), 7.50-7.54 (m, 1H), 7.29-7.39 (m, 2H), 3.96 (s, 2H) ppm. ^{1}H NMR (400MHz, Pyridine-d_5): δ = 7.95-7.99 (m, 2H), 7.88-7.93 (m, 3H), 7.82 (br. s., 1H), 7.74 (d, J=8.2 Hz, 1H), 7.68 (br. d, J=2.0 Hz, 1H), 7.61 (br. dd, J=8.2, 2.0 Hz, 1H), 7.42 (td, J=7.4, 1.2 Hz, 1H), 7.37 (td, J=7.5, 1.4 Hz, 1H), 3.86 (s, 2H) ppm. ^{13}C NMR (126 MHz, Pyridine-d_6): δ = 37.38, 120.81, 121.54, 121.65, 122.49, 123.28, 124.63, 125.48, 125.68, 126.42, 129.27, 130.88, 134.03, 140.29, 141.07, 141.75, 141.94, 144.80, 145.29, 146.67, 150.35 ppm. HRMS (EI) calcd for $C_{21}H_{13}BrS$ $[M]^+$ 375.9921, found 375.9912.

2-Bromo-7-(S,S-dioxo-1-benzothiophen-2-yl)-fluorene (10-BTh(O_2),Br):

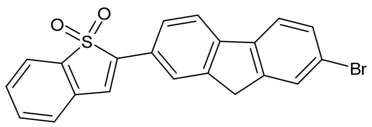

Compound **10**-BTh(O_2),Br was prepared similar to published protocol [17,24]. The mixture of 7-(benzthiophen-2-yl)-fluorenyl bromide **10**-BTh,Br (400 mg, 1.06 mmol, 1 eq), 100% MCPBA (1.14 g, 6.63 mmol, 6.25 eq) and CH_2Cl_2 (20 mL) was stirred at ambient temperature for 14 h. The reaction mixture was diluted with CH_2Cl_2 (40 mL), washed with sat. aq. $NaHCO_3$ solution (30 mL), water (2×40 mL) and brine (40 mL). The organic solution was dried over Na_2SO_4 and 4.5 g of SiO_2 added to prepare a dry load. Flash chromatography was performed on a 25 g cartridge. Elution with 20% CH_2Cl_2 in *n*-hexane (1CV), gradient 20-100% CH_2Cl_2 in *n*-hexane (10CV), CH_2Cl_2 (1CV), gradient 0-100% of MeOH in CH_2Cl_2 (5CV). The major fraction (R_f = 0.75 in CH_2Cl_2) was collected. After evaporation of solvents and drying, compound **10**-BTh(O_2),Br (235 mg, 54%) was isolated as a yellowish solid.

^{1}H NMR (400 MHz $CDCl_3$): δ = 8.03 (m, 1H), 7.81-7.84 (m, 2H), 7.77-7.80 (m, 1H), 7.70-7.72 (m, 1H), 7.66 (d, *J* = 8.2 Hz, 1H), 7.55-7.59 (m, 1H), 7.48-7.55 (m, 2H), 7.40 (d, *J* = 7.4 Hz, 1H), 7.31 (d, *J* = 0.8 Hz, 1H), 3.95 (s, 2H) ppm. ^{1}H NMR (500 MHz $CDCl_3$): δ = 8.04 (br s, 1H), 7.81-7.83 (m, 2H), 7.79 (d, *J* = 7.5 Hz, 1H), 7.72 (d, *J* = 0.7 Hz, 1H), 7.67 (d, *J* = 8.1 Hz, 1H), 7.58 (td, *J* = 7.6, 0.9 Hz, 1H), 7.53 (dd, *J* = 8.1, 1.6 Hz, 1H), 7.50 (td, *J* = 7.4, 0.8 Hz, 1H), 7.42 (d, *J* = 7.5 Hz, 1H), 7.32 (s, 1H), 3.96 ppm (s, 2H). ^{13}C NMR (126 MHz, $CDCl_3$): δ = 145.8, 143.7, 142.9, 142.8, 139.7, 137.0, 133.8, 131.3, 130.2, 129.8, 128.5, 125.8, 125.7, 124.9, 123.1, 123.1, 121.7, 121.6, 121.6, 120.7, 36.8 ppm. HRMS (EI) calcd for $C_{21}H_{13}BrO_2S$ $[M]^+$ 407.9820, found 407.9814.

2-(Thiophen-2-yl)-7-(4-pyridyl)-fluorene (11-Py,Th):

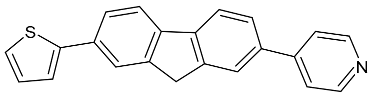

According to **GP3** and using 2-bromofluorene **10**-Py,Br (480 mg, 1.49 mmol, 1.0 eq) and boronic ester **2c** (470 mg, 2.24 mmol, 1.5 eq), a greyish solid material was isolated, and characterized as compound **11**-Py,Th (248 mg, 51%).

^{1}H NMR (400 MHz, $CDCl_3$): δ = 8.65-8.72 (m, *J* = 5.9 Hz, 2H), 7.89 (d, *J* = 7.8 Hz, 1H), 7.84 (dd, *J* = 8.2, 0.8 Hz, 1H), 7.82 (d, *J* = 7.8 Hz, 1H), 7.69-7.72 (m, 1H), 7.67-7.69 (m, 1H), 7.62-7.66 (m, *J* = 6.3 Hz, 2H), 7.39 (dd, *J* = 3.5, 1.2 Hz, 1H), 7.31 (dd, *J* = 5.1, 1.2 Hz, 1H), 7.12 (dd, *J* = 5.3, 3.7 Hz, 1H), 4.03 (s, 2H) ppm. ^{1}H NMR (500 MHz, DMSO-d_6): δ = 8.80 (br. d, *J*=5.9 Hz, 2H), 8.19 (br. d, *J*=0.9 Hz, 1H), 8.14 (br. d, *J*=6.3 Hz, 2H), 8.12 (d, *J*=7.9 Hz, 1H), 8.04 (d, *J*=7.8 Hz, 1H), 8.00 (br. dd, *J*=8.0, 1.7 Hz, 1H), 7.95 (br. dd, *J*=1.6, 0.6 Hz, 1H), 7.76 (br. dd, *J*=7.9, 1.8 Hz, 1H), 7.62 (dd, *J*=3.6, 1.1 Hz, 1H), 7.58 (dd, *J*=5.1, 1.1 Hz, 1H), 7.18 (dd, *J*=5.1, 3.6 Hz, 1H), 4.11 (s, 2H) ppm. ^{13}C NMR (126 MHz, DMSO-d_6): δ = 151.4, 146.2, 144.9, 144.4, 143.6, 143.0, 139.5, 134.0, 133.2, 128.6, 126.6, 125.8, 124.5, 124.2, 123.9, 122.3, 122.2, 121.4, 121.0, 36.6 ppm. HRMS (EI) calcd for $C_{22}H_{15}NS$ $[M]^+$ 325.0925, found 325.0916.

2-(1-Thiophen-2-yl)-7-(4-pyridyl)-fluorene (11-BTh,Py):

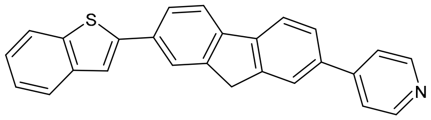

According to **GP3** and using of 2-bromofluorene derivative **10**-BTh,Br (100 mg, 0.265 mmol, 1.0 eq) and pyridyl boronic acid ester **2b** (87 mg, 0.424 mmol, 1.6 eq), compound **11**- BTh,Py (90.9 mg, 51%) was isolated as a greyish solid. To dissolve the NMR sample was prepared by addition of 20 µL of TFA to the mixture of 0.5 mL DMSO-d_6 and 1 mg of the title compound.

^{1}H NMR (500 MHz, DMSO-d_6): δ = 8.95-9.00 (m, *J* = 6.9 Hz, 2H), 8.50-8.54 (m, *J* = 7.1 Hz, 2H), 8.34 (d, *J* = 1.0 Hz, 1H), 8.20 (d, *J* = 8.0 Hz, 1H), 8.15 (s, 1H), 8.13 (s, 1H), 8.07 (d, *J* = 1.0 Hz, 1H), 7.97-7.99 (m, 1H), 7.97 (s, 1H), 7.90 (dd, *J* = 8.0, 1.7 Hz, 1H), 7.86 (s, 1H), 7.34-7.42 (m, 2H), 4.17 (s, 2H) ppm. ^{13}C NMR (126 MHz, DMSO-d_6): δ = 156.3, 145.4, 144.9, 144.3, 143.5, 142.0 (2C), 140.7, 140.3, 138.8, 133.4, 132.6, 127.5, 125.4, 125.0 (2C), 124.9, 123.9, 123.7 (2C), 123.0, 122.6, 121.9, 121.5, 120.5, 36.8 ppm. HRMS (ESI) calcd for $C_{26}H_{17}NS$ $[M+H]^+$ 376.1154, found 376.1157.

2-(S,S-Dioxo-1-benzothiophen-2-yl)-7-(4-pyridyl)-fluorene (11-BTh(O_2),Py)

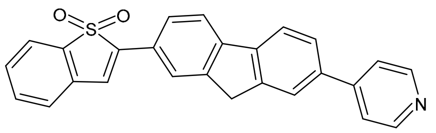

According to **GP3** and using 2-bromofluorene derivative **10**-BTh(O_2),Br (50 mg, 0.122 mmol, 1.0 eq) and pyridyl boronic acid ester **2b** (35 mg, 0.17 mmol, 1.4 eq), solid material **11**-BTh(O_2),Py (40.5 mg, 81%) was isolated.

^{1}H NMR (500 MHz, $CDCl_3$): δ = 8.72-8.76 (m, 2H), 8.16 (br s, 1H), 8.10-8.13 (m, 2H), 8.03 (d, *J* = 7.9 Hz, 1H), 7.99 (br s, 1H), 7.98 (d, *J* = 8.3 Hz, 1H), 7.91 (dd, *J* = 8.0, 1.5 Hz, 1H), 7.83 (dd, *J* = 8.1, 1.7 Hz, 1H), 7.81 (d, *J* = 8.0 Hz, 1H), 7.62 (td, *J* = 7.6, 1.1 Hz, 1H), 7.54 (td, *J* = 7.6, 0.9 Hz, 1H), 7.46 (d, *J* = 7.3 Hz, 1H), 7.40 (d, *J* = 0.5 Hz, 1H), 4.13 (s, 2H) ppm. ^{13}C NMR (126 MHz, $CDCl_3$): δ = 145.5, 144.9, 144.8, 142.6, 142.0, 141.14, 141.13, 141.08, 137.0, 133.9, 131.1, 130.1, 127.2, 127.1, 126.0, 125.1, 124.5, 123.9, 123.7, 123.3, 121.8, 121.7, 121.7, 37.1 ppm. HRMS (ESI) calcd for $C_{26}H_{17}NO_2S$ $[M+H]^+$ 408.1053, found 408.1053.

2-{4-[7-(S,S-Dioxo-1-benzothiophen-2-yl)-fluoren-2-yl]pyridin-1-ium-1-yl}-4,4,5,5-tetrafluoro-3-oxocyclopent-1-en-1-olate (11-BTh(O_2),Py-betaine):

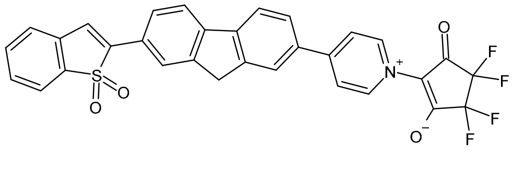

According to **GP5** and using compound **11**-BTh(O_2),Py, **11**-BTh(O_2),Py-betaine was isolated as a yellow solid (26 mg, 90%). LCMS analysis (standard method see experimental details avobe) gave major peak with R_t = 7.7 min, m/z=574.00 (negative mode) 576.28 (positive mode), to be **11**-BTh(O_2),Py-betaine $C_{31}H_{17}F_4NO_4S$ (M 575.08). Due to low solubility in common solvents, ^{13}C NMR was not recorded for this compound.

^{1}H NMR (400 MHz, DMSO-d_6): δ = 9.26-9.32 (m, J = 7.0 Hz, 2H), 8.62-8.68 (m, J = 7.0 Hz, 2H), 8.42 (s, 1H), 8.20-8.29 (m, 3H), 8.16 (s, 1H), 8.11 (s, 1H), 7.95-8.00 (m, 2H), 7.73-7.77 (m, 1H), 7.64-7.70 (m, 2H), 4.23 (br s, 2H) ppm. ^{19}F NMR (376 MHz, DMSO-d_6): δ = -126.0 (s, 4F) ppm.

2-(4-Pyridyl)-7-(4-dimethylaminophenyl)-fluorene (11-Py,DmP):

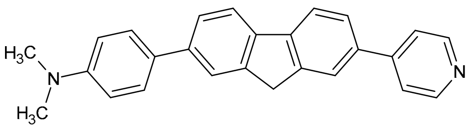

According to **GP3** and with heating at 100 °C for 16 h, using 2-bromofluorene derivative **10**-DmP,Br (70 mg, 0.192 mmol, 1.0 eq) and pinacol boronic ester **2b** (53.2 mg, 0.24 mmol, 1.35 eq) the formed precipitate was collected and washed with water. The organic phase was evaporated to give an additional amount of material. Both portions were combined and dried to give 80 mg of solid material. ^{1}H NMR was taken from this sample. The solid was dissolved in CH_2Cl_2/MeOH (5/1, 150 mL), and co-evaporated with 0.8 g of silica gel (dry load). Flash chromatography was performed on a 5 g cartridge. Elution with 40% CH_2Cl_2 in n-hexane (1CV), gradient 40-100% CH_2Cl_2 in n-hexane (10CV), CH_2Cl_2 (1CV), gradient 0-100% of MeOH in CH_2Cl_2 (10CV). The fractions containing the product (TLC, R_f = 0.50 in CH_2Cl_2/MeOH – 10/1) were evaporated and dried to give target compound **11**-Py,DmP (36 mg 51%).

^{1}H NMR (400 MHz, $CDCl_3$): δ = 8.67-8.71 (m, 2H), 7.92 (d, J = 7.8 Hz, 1H), 7.89 (br d, J = 1.2 Hz, 1H), 7.87 (d, J = 8.2 Hz, 1H), 7.83 (m, J = 6.7 Hz, 2H), 7.79 (d, J = 0.8 Hz, 1H), 7.74 (dd, J = 8.0, 1.8 Hz, 1H), 7.64 (dd, J = 8.0, 1.8 Hz, 1H), 7.57-7.62 (m, 2H), 6.84-6.93 (m, 2H), 4.05 (s, 2H), 3.03 (s, 6H) ppm. HRMS (ESI) calcd for $C_{26}H_{22}N_2$ $[M+H]^+$ 363.1856, found 363.1853.

2-(Thiophen-2-yl)-7-(4-dimethylaminophenyl)-fluorene (11-Th,DmP):

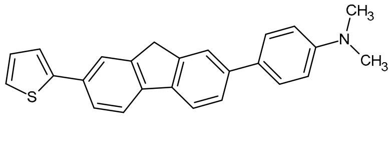

This compound was prepared according to **GP3**, and using 2-bromofluorene derivative **10**-Th,Br (68 mg, 0.18 mmol, 1.0 eq) and 4-dimethylaminophenyl boronic acid ester **2f** (70 mg, 0.28 mmol, 1.5 eq).

The reaction mixture (3 mL) was diluted with EtOAc (3 mL), shaken and the formed solid was filtered off, washed with toluene/*n*-hexane mixture (1/1, 2×3 mL) mixture, and water (2×3 mL). After drying, the solid material **12**-Th,DmP (26 mg, 38%) was isolated.

^{1}H NMR (400 MHz, $CDCl_3$): δ = 7.74-7.81 (m, 4H), 7.57-7.67 (m, 4H), 7.37 (dd, *J*=3.6, 0.9 Hz, 1H), 7.28 (dd, *J*=5.0, 0.9 Hz, 1H), 7.10 (dd, *J*=5.0, 3.6 Hz, 1H), 6.49-7.08 (m, 2H), 3.99 (s, 2H), 3.05 (br. s., 6H) ppm. HRMS (ESI) calcd for $C_{25}H_{21}NS$ $[M+H]^+$ 368.1467, found 368.1471.

2-(1-Benzothiophen-2-yl)-7-(4-dimethylaminophenyl)-fluorene (11-BTh,DmP):

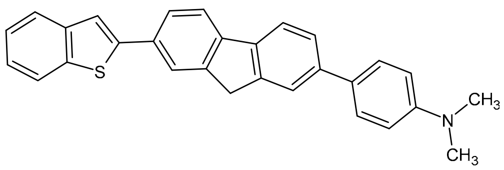

According to **GP3**, and using 2-bromofluorene derivative **10**-BTh,Br (101 mg, 0.27 mmol, 1.0 eq) and 4-dimethylaminophenyl boronic acid ester **2f** (108 mg, 0.44 mmol, 1.6 eq), solid **11**-BTh,DmP (104 mg 92%) was isolated.

^{1}H NMR (400 MHz, CD_2Cl_2): δ = 7.93-7.95 (m, 1H), 7.79-7.88 (m, 6H), 7.65 (d, *J*=0.8 Hz, 1H), 7.58-7.60 (m, *J*=9.0 Hz, 2H), 7.32-7.39 (m, 3H), 6.82-6.86 (m, *J*=9.0 Hz, 2H), 4.04 (s, 2H), 3.01 (s, 6H) ppm. ^{13}C NMR (126 MHz, CD_2Cl_2): δ = 128.1, 125.9, 125.6, 125.1, 124.8, 124.0, 123.5, 123.2, 122.8, 120.8, 120.6, 119.6, 113.3, 40.9, 37.6, (quaternary C signals were not resolved) ppm. HRMS (ESI) calcd for $C_{29}H_{23}NS$ $[M+H]^+$ 418.1624, found 418.1627.

2-(1-Benzothiophen-2-yl)-7-(*N*,*N*,*N*-trimethylanilinium-4-yl)-fluorene iodide (11-BTh,DmP-Me$_3$N$^+$):

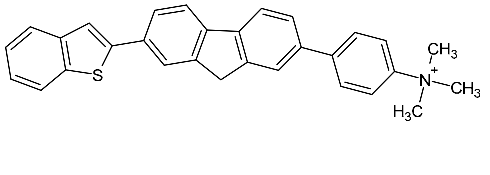

The titles compound **12**-BTh,DmP-Me_3N^+ was prepared according to protocol [25] and using MeCN as solvent instead of dioxane. The compound **12**-BTh,DmP (20 mg, 48 µmol, 1.0 eq) was mixed with MeCN (3 mL) and MeI (1.36 g, 9.58 mmol, 200 eq) in a 12 mL pressure tube and stirred under heating

at 100 °C for 19 h. The reaction mixture was cooled, filtered through the paper filter, and the solid material was dried to give ammonium iodide **12**-BTh,DmP-Me_3N^+ (9.2 mg, 34%). Its optical properties were measured without further purification.

^1H NMR (500 MHz, DMSO-d_6): δ = ppm 8.01 - 8.10 (m, 8 H) 7.99 - 8.01 (m, 1 H) 7.96 (br. s, 1 H) 7.87 (m, 2 H) 7.82 (dd, *J*=7.90, 1.74 Hz, 1 H) 7.35 - 7.44 (m, 2 H) 4.13 (s, 2 H) 3.66 (s, 9 H) ppm. ^{13}C NMR (126 MHz, DMSO-d_6): δ = 146.3, 144.6, 144.5, 143.6, 141.9, 140.9, 140.8, 140.6, 138.6, 136.9, 132.4, 128.0, 126.1, 125.2, 124.9, 124.7, 123.82, 123.77, 122.9, 122.5, 121.1, 121.1, 121.0, 120.0, 56.5, 36.7 ppm.

HRMS (ESI) calcd for $C_{30}H_{26}NS$ $[M]^+$ 432.1780, found 432.1784.

2-(S,S-Dioxo-1-benzothiophen-2-yl)-7-(4-dimethylaminophenyl)-fluorene (11-BTh(O₂),DmP):

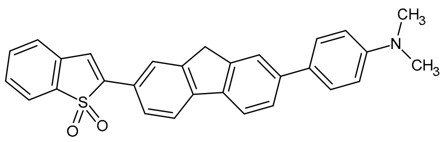

According to **GP3**, and using 2-bromofluorene **10**-BTh(O_2),Br (50 mg, 0.12 mmol, 1.0 eq) as well as 4-dimethylaminophenyl boronic acid ester **2f** (36 mg, 0.15 mmol, 1.2 eq), compound **11**-BTh(O_2),DmP (50 mg, 91%) was isolated.

^1H NMR (400 MHz, DMSO-d_6): δ = 8.05 - 8.10 (m, 2 H) 8.03 (br. d, *J*=0.80 Hz, 1 H) 7.99 (d, *J*=8.22 Hz, 1 H) 7.95 (br. d, *J*=7.40 Hz, 1 H) 7.91 (br. dd, *J*=8.20, 1.60 Hz, 1 H) 7.86 (br. s, 1 H) 7.75 (td, *J*=7.40, 1.20 Hz, 1 H) 7.62 (m, *J*=9.00 Hz, 5 H) 6.83 (d, *J*=9.00 Hz, 2 H) 4.09 (s, 2 H) 2.96 (s, 6 H) ppm. HRMS (ESI) calcd for $C_{29}H_{23}NO_2S$ $[M+H]^+$ 450.1522, found 450.1526.

2-(S,S-Dioxo-1-benzothiophen-2-yl)-7-(*N*,*N*,*N*-trimethylanilinium-4-yl)-fluorene trifluoroacetate (11-BTh(O₂),DmP-Me₃N⁺):

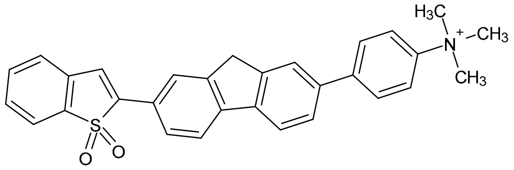

The titled compound **11**-BTh(O_2),DmP-Me_3N^+ was prepared according to protocol [21] and using MeCN as solvent instead of dioxane. The mixture of compound **11**-BTh(O_2),DmP (8.0 mg, 18 µmol, 1.0 eq), MeCN (2 mL) and MeI (131 mg, 0.925 mmol, 51 eq) in a 12 mL pressure tube was stirred under heating at 85 °C for 14 h. Then the solvents were removed under reduced pressure. The formed yellowish precipitate (10 mg) was dissolved in DMSO (1 mL) and loaded onto reverse phase silica gel cartridge (25 g) for Biotage Isolera One MPLC system. Elution with 15% MeCN (0.1% TFA) in H_2O (0.1% TFA) (1CV), gradiend 15-95% MeCN (0.1% TFA) in H_2O (0.1% TFA) (10CV).The major fraction was freeze-dried to give **11**-BTh(O_2),DmP-Me_3N^+ trifluoroacetate salt (1.5 mg, 15%).

^{1}H NMR (600 MHz, DMSO-d_6): δ = 8.17 (d, *J*=8.1 Hz, 1H), 8.10-8.14 (m, 2H), 8.06-8.09 (m, 3H), 8.02-8.06 (m, 3H), 7.93-7.98 (m, 2H), 7.85 (br. d, *J*=8.0 Hz, 1H), 7.76 (td, *J*=7.4, 0.8 Hz, 1H), 7.68 (br. d, *J*=7.5 Hz, 1H), 7.64 (td, *J*=7.5, 0.8 Hz, 1H), 4.16 (br. s, 2H), 3.66 (s, 9H) ppm. ^{13}C NMR (126 MHz, DMSO-d_6): δ = 146.3, 144.8, 144.4, 142.5, 141.7, 141.2, 140.4, 137.4, 136.3, 134.5, 130.9, 130.4, 128.0, 126.2, 125.8, 125.6, 125.4, 124.6, 123.8, 122.7, 121.4, 121.3, 121.1, 56.4, 45.7, 36.7 ppm. HRMS (ESI) calcd for $C_{30}H_{26}NO_2S$ $[M]^+$ 464.1673, found 464.1682.

2-(Morpholin-4-yl)-7-(pyridin-4-yl)-fluorene (12):

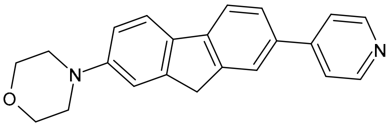

According to **GP4**, and starting from 2-bromofluorene **10**-Py,Br (69 mg, 0.22 mmol), 141 mg of solid brick-red compound was isolated. Flash chromatography was performed on a 10 g cartridge. Elution with 20% CH_2Cl_2 in *n*-hexane (1CV), gradient 20-100% CH_2Cl_2 in *n*-hexane (8CV), CH_2Cl_2 (1CV), gradient 0-100% of MeOH in CH_2Cl_2 (7CV). After evaporation and drying of the fractions with product (TLC, R_f=0.75 in CH_2Cl_2), compound **12** was isolated as a pink-red solid (57.2 mg, 81%).

^{1}H NMR (400 MHz, $CDCl_3$): δ = 8.79-8.83 (m, *J* = 7.0 Hz, 2H), 8.04-8.07 (m, *J* = 7.0 Hz, 2H), 7.90 (d, *J* = 1.2 Hz, 1H), 7.86 (d, *J* = 7.8 Hz, 1H), 7.79 (d, *J* = 8.6 Hz, 1H), 7.77 (dd, *J* = 8.2, 2.0 Hz, 1H), 7.29 (d, *J* = 1.6

Hz, 1H), 7.12 (dd, J = 8.4, 2.2 Hz, 1H), 4.00 (s, 2H), 3.94-3.99 (m, 4H), 3.30-3.36 (m, 4H) ppm. HRMS (ESI) calcd for $C_{22}H_{20}N_2O$ $[M+H]^+$ 329.1648, found 329.1651.

4,4,5,5-Tetrafluoro-2-{4-[7-(morpholin-4-ium-4-yl)-fluoren-2-yl]pyridin-1-ium-1-yl}-3-oxocyclopent-1-en-1-olate trifluoroacetate (13):

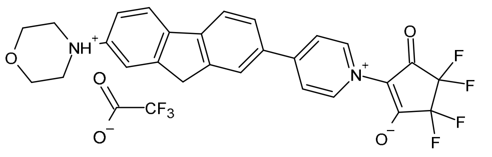

According to **GP5**, and using compound **12** (12 mg, 39 µmol), 17.9 mg of titled compound was isolated. Purification was performed by preparative HPLC, and after freeze drying of the major fraction with R_t=7.1 min according to LCMS analysis (standard method, see general experimental details), compound **13** (7.6 mg, 39%) was isolated.

^{1}H NMR (600 MHz, DMSO-d_6): δ = 9.20-9.23 (m, J=7.3 Hz, 2H), 8.57-8.60 (m, J=7.3 Hz, 2H), 8.29 (dd, J=1.8, 0.7 Hz, 1H), 8.13 (dd, J=8.1, 1.8 Hz, 1H), 7.99 (d, J=8.1 Hz, 1H), 7.89 (d, J=8.5 Hz, 1H), 7.25 (br. d, J=2.2 Hz, 1H), 7.08 (dd, J=8.5, 2.2 Hz, 1H), 4.00 (br. s, 2H), 3.76-3.79 (m, 4H), 3.23-3.25 (m, 4H) ppm.

^{13}C NMR (126 MHz, DMSO-d_6): δ = 158.1 (dm, $^2J_{C-F}$=36.0 Hz, 2C), 154.1, 151.7, 146.11, 146.06, 143.7, 141.9, 131.0, 129.8, 127.5, 124.5, 122.8, 121.9, 119.9, 116.1 (d, $^3J_{C-F}$=11.0 Hz, 1C, HMBC cross peak with protons 9.21 ppm), 114.4, 111.3, 66.1, 48.3, 36.6 ppm. ^{19}F NMR (376 MHz, DMSO-d_6): δ = -74.7 (br. s., 3F, CF_3COO^-), -126.0 ppm (s, 4F). HRMS (ESI) calcd for $C_{27}H_{21}F_4N_2O_3$ $[M+H]^+$ 497.1483 found 497.1482.

2-Bromo-7-(4-pyridyl)-fluoren-9-one (15):

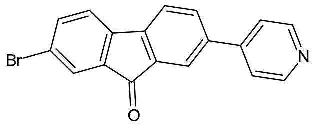

The reaction was performed according to **GP2** using 2-bromo-7-iodo-fluorenone (**14**) (1.50 g, 3.90 mmol, 1.0 eq) with pinacol boronic ester **2b** (879 mg, 4.30 mmol, 1.1 eq), Na_2CO_3 (1.447 g, 13.65 mmol, 3.5 eq) and $Pd(PPh_3)_4$ (120 mg, 0.105 mmol, 0.027 eq) in a 100 mL Schlenk flask. Toluene (18 mL),

water (18 mL) and Aliquat 336 (80 mg) were added and the mixture was degassed by bubbling argon gas for 5 min. The reaction time was 15 h at 100 °C, during which a yellow precipitate was formed. The formed suspension was filtered, and the residue washed with toluene (2×10 mL) and water (3×10 mL), providing, after drying, compound **15** (0.655 g 50%).

^{1}H NMR (500 MHz, $CDCl_3$): δ = 8.71 (d, *J*=6.3 Hz, 2H), 7.95 (dd, *J*=1.8, 0.6 Hz, 1H), 7.82 (dd, *J*=1.8, 0.4 Hz, 1H), 7.81 (dd, *J*=7.8, 1.9 Hz, 1H), 7.67 (dd, *J*=7.9, 1.9 Hz, 1H), 7.65 (dd, *J*=7.8, 0.6 Hz, 1H), 7.57 (d, *J*=6.3 Hz, 2H), 7.47 (dd, *J*=7.9, 0.4 Hz, 1H) ppm. ^{13}C NMR (126 MHz, $CDCl_3$): δ = 191.7, 149.9, 147.4, 144.3, 142.3, 139.2, 137.5, 136.0, 134.7, 133.6, 127.9, 123.6, 123.1, 122.1, 121.3, 121.2 ppm. HRMS (ESI) calcd for$C_{27}H_{19}N_3O_2S$ $[M+H]^+$ 336.0019, found 336.0018.

2-(Morpholin-4-yl)-7-(pyridin-4-yl)-fluoren-9-one (16):

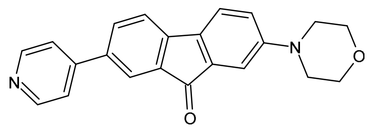

According to **GP4**, and using 2-bromo-7-(4-pyridyl)-fluorenone (**15**) (300 mg, 0.89 mmol) the reaction mixture was heated at 90 °C for 16 h. The formed precipitate was separated by centrifugation, washed with dioxane, water and freeze-dried at 0.2 mbar to give product **16** (300 mg, 98%) as a reddish solid.

^{1}H NMR (400 MHz, DMSO-d_6): δ = 8.57-8.69 (m, 2H), 8.00 (dd, *J* = 7.8, 2.0 Hz, 1H), 7.91 (d, *J* = 1.6 Hz, 1H), 7.77-7.80 (m, 2H), 7.77 (d, *J* = 7.8 Hz, 1H), 7.69 (d, *J* = 8.6 Hz, 1H), 7.21 (d, *J* = 2.3 Hz, 1H), 7.16 (dd, *J* = 8.6, 2.3 Hz, 1H), 3.68-3.81 (m, 4H), 3.19-3.28 (m, 4H) ppm. ^{13}C NMR (126 MHz, DMSO-d_6): δ = 193.2, 152.5, 150.3, 145.8, 145.6, 136.4, 135.2, 134.4, 133.9, 133.3, 122.5, 122.0, 120.8, 120.8, 120.2, 110.3, 65.9, 47.9 ppm. HRMS (ESI) calcd for $C_{22}H_{19}N_2O_2$ $[M+H]^+$ 343.1441 found 343.1444.

4,4,5,5-Tetrafluoro-2-{4-[7-(morpholin-4-yl)-9-oxofluoren-2-yl]pyridin-1-ium-1-yl}-3-oxocyclopent-1-en-1-olate (17):

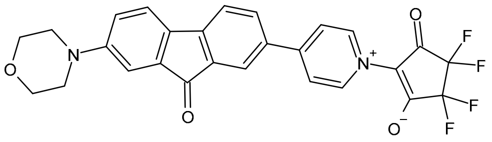

According to **GP5**, and starting with compound **16** (100 mg, 0.292 mmol), betaine **17** (91 mg, 60%) was obtained.

^{1}H NMR (400 MHz, DMSO-d_6): δ = 9.22-9.29 (m, *J* = 7.4 Hz, 2H), 8.60-8.67 (m, *J* = 7.4 Hz, 2H), 8.31 (dd, *J* = 8.0, 1.8 Hz, 1H), 8.24 (d, *J* = 1.6 Hz, 1H), 7.90 (d, *J* = 8.2 Hz, 1H), 7.78 (d, *J* = 8.6 Hz, 1H), 7.26 (d, *J* = 2.3 Hz, 1H), 7.21 (dd, *J* = 8.4, 2.5 Hz, 1H), 3.70-3.81 (m, 4H), 3.25-3.29 (m, 4H) ppm. ^{19}F NMR (376 MHz, DMSO-d_6): δ = -126.0 (s, 4F) ppm. HRMS (ESI) calcd for $C_{27}H_{18}$ $F_4N_2O_4Na$ $[M+Na]^+$ 533.1095 found 533.1107.

(E/Z)-2-(Morpholin-4-yl)-7-(pyridin-4-yl)-fluoren-9-one mesylhydazone (18)

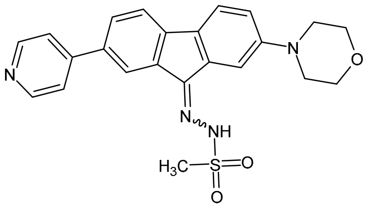

Fluorenone **16** (136 mg, 0.397 mmol, 1.0 eq), mesylhydazide hydrohloride [19] (192 mg, 0.616 mmol, 3.3 eq) and EtOH (20 mL) were placed in a 250 mL round bottom flask connected with a cartridge with holes containing molecular seaves 4Å (see photo). The reaction mixture was carefully stirred at 44 °C for 62 h. Then it was cooled down to ambient temperature and the reddish precipitate was filtered off. The collected solid was washed with EtOH (4 mL), CH_2Cl_2 (4 mL) and dried under 0.4 mbar to provide product **18** (140 mg, 81%). NOTE: The reaction without the drying cartridge did not complete giving a mixture of product **18** and starting ketone **16**.

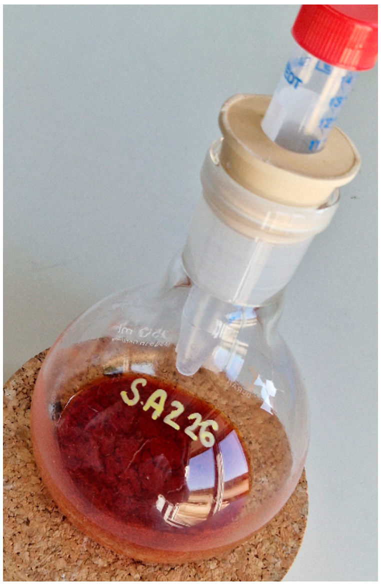

^{1}H NMR (400 MHz, DMSO-d_6, *E/Z* mixture), A is major isomer: δ = 8.93-8.99 (m, 2H, A), 8.86-8.93 (m, *J* = 6.7 Hz, 1H, B), 8.51 (d, *J* = 1.6 Hz, 1H, A), 8.38-8.44 (m, *J* = 7.0 Hz, 1H, B), 8.33-8.38 (m, *J* = 6.7 Hz, 2H, A), 8.25 (d, *J* = 1.6 Hz, 1H, B), 8.06-8.13 (m, 1H, A+B), 7.94 (d, *J* = 8.2 Hz, 1H, A), 7.93 (d, *J* = 7.8 Hz, 1H, B), 7.87 (d, *J* = 8.6 Hz, 1H, B), 7.79 (d, *J* = 8.6 Hz, 1H, A), 7.69 (d, *J* = 2.0 Hz, 1H, B), 7.35 (d, *J* = 2.3 Hz, 1H, A), 7.19 (dd, *J* = 8.6, 2.3 Hz, 1H, B), 7.11 (dd, *J* = 8.6, 2.3 Hz, 1H, A), 3.72-3.83 (m, 6H, A+B), 3.30 (s, 2H, B), 3.28 (s, 3H, A), 3.26 (br d, *J* = 4.7 Hz, 6H, A+B) ppm.

(*E/Z*)-4,4,5,5-Tetrafluoro-2-(4-{9-[2-(methanesulfonyl)hydrazinylidene]-7-(morpholin-4-yl)-fluoren-2-yl}pyridin-1-ium-1-yl)-3-oxocyclopent-1-en-1-olate (19):

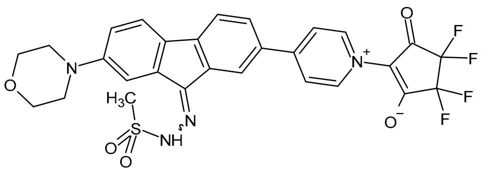

Product **19** was prepared using DMSO as a solvent as reported in literature [26]. Compound **18** (136 mg, 0.313 mmol, 1.0 eq) was dissolved in anhydrous DMSO (10 mL) in a 25 mL "pressure tube". Argon was bubbled through the solution for about 10 min, and then it was cooled to 0 °C. Perfluorocyclopentene (334 µL, 531 mg, 2.5 mmol, 8 eq) was added as described in **GP5**, and the tube was stopped. The reaction mixture was stirred at ambient temperature for 15 h. The bottle was opened and 45 µL of water (3-4 drops) were added. The solution was stirred for 1.5 h and then freeze-dried at 0.01-0.02 mbar to give product **19** (180 mg, 95%).

^{1}H NMR (600 MHz, DMSO-d_6, *E/Z* mixture), A is major isomer: δ = 11.39 (br s, 1H), 11.22 (br s, 1H, A), 9.28-9.31 (m, 2H), 9.23-9.27 (m, 2H, A), 8.63-8.66 (m, 2H, A), 8.54-8.57 (m, 2H), 8.53 (d, *J* = 1.5 Hz, 1H), 8.35 (d, *J* = 1.4 Hz, 1H, A), 8.20 (dd, *J* = 8.0, 1.8 Hz, 1H), 8.18 (dd, *J* = 8.1, 1.8 Hz, 1H, A), 7.98 (d, *J* = 8.0 Hz, 1H), 7.96 (d, *J* = 8.0 Hz, 1H, A), 7.90 (d, *J* = 8.4 Hz, 1H, A), 7.81 (d, *J* = 8.4 Hz, 1H), 7.68 (d, *J* = 2.2 Hz, 1H, A), 7.36 (d, *J* = 2.3 Hz, 1H), 7.20 (dd, *J* = 8.6, 2.2 Hz, 1H, A), 7.12 (dd, *J* = 8.6, 2.5 Hz, 1H), 3.79-3.82 (m, 4H, A), 3.76-3.78 (m, 4H), 3.31 (s, 3H, A), 3.29 (s, 3H), 3.26-3.30 (m, 8H) ppm. ^{13}C NMR (126 MHz, DMSO-d_6), A is major isomer: δ = 171.64 (t, $^2J_{CF}$ = 25.3 Hz, 1C, A), 171.57 (t, $^2J_{CF}$ = 26.8 Hz, 1C), 153.7,

153.3, 152.5, 152.2, 150.3, 149.7, 145.7, 143.7, 142.4 (2C), 141.8(2C, A), 138.6, 136.9, 132.2, 131.6, 131.4, 131.3, 131.0 (1C, A), 130.9, 129.9, 129.0, 126.9 (1C, A), 123.4 (2C, A+B), 122.7 (1C, A), 122.5, 120.8, 120.5, 120.4 (1C, A), 117.9 (1C, A), 117.1, 114.4 (1C, A), 109.6, 107.3, 65.99 (2 C), 65.96 (2C, A), 48.2 (2C, A), 48.0 (2 C), 37.8, 37.5 (1C, A) ppm.

2-{4-[9-Diazo-7-(morpholin-4-yl)-fluoren-2-yl]pyridin-1-ium-1-yl}-4,4,5,5-tetrafluoro-3-oxocyclopent-1-en-1-olate (20):

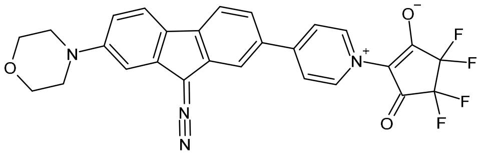

Compound **20** was prepared using mesylhydrazone hydrochloride in close analogy to the reported procedure [20]. Betaine **19** (50 mg, 83 µmol, 1.0 eq) was dissolved in THF (25 mL) and the solution passed thought a pad of silica gel, which was washed with THF to collect all the coloured liquid. The solvent was removed, and the residue dried to give 49.5 mg of compound **19**. This was re-dissolved in dry THF (25 mL), diisopropylethylamine (140 µL, 0.83 mmol, 10 eq) added and the mixture stirred at 67 °C for 3 h. According to LCMS of the reaction mixture, the following compounds were formed (HPLC areas): starting **19** (24%), ketone **17** (22%), diazocompound **20** (54%). The reaction mixture was colled, concentrated to 3 mL and loaded onto an equilibrated cartridge (Sfär, 25 g of 60 µm silica gel). Elution was performed with gradient 30-100% EtOAc in hexanes (10CV), EtOAc (4CV), THF (100 mL), THF/EtOH (1/1, 100 mL) to collect coloured fractions. The first colored fraction eluted with pure EtOAc was evaporated, affording, after drying, diazocompound **20** (6.1 mg, 14%).

^{1}H NMR (400 MHz, DMSO-d_6): δ = 9.17-9.24 (m, 2H), 8.61-8.67 (m, 2H), 8.51 (d, *J* = 1.6 Hz, 1H), 8.13 (d, *J* = 8.2 Hz, 1H), 8.03 (d, *J* = 8.6 Hz, 1H), 8.02 (dd, *J* = 8.4, 1.8 Hz, 1H), 7.33 (d, *J* = 2.3 Hz, 1H), 7.05 (dd, *J* = 8.8, 2.2 Hz, 1H), 3.72-3.83 (m, 4H), 3.26-3.31 (m, 4H) ppm. ^{13}C NMR (101 MHz, DMSO-d_6): δ = 154.1, 151.5, 142.0, 135.8, 134.5, 133.2, 129.6, 129.2, 124.4, 123.0, 122.9, 121.6, 120.7, 119.4, 113.2, 105.1, 66.1, 48.3 ppm; due to C-F coupling the signals of the betaine part were not resolved. ^{19}F NMR (376

MHz, DMSO-d_6): δ = -126.0 (s, 1F) ppm. HRMS (ESI) calcd for $C_{27}H_{18}F_4N_2O_3$ $[M+H]^+$ 523.1388 found 523.1395. IR (KBr pellet): 3422, 2064 CN_2, 1630, 1599, 1433, 1325, 1221, 1122, 1061 cm^{-1}.

2-[4-(7-Bromo-9-oxofluoren-2-yl)pyridin-1-ium-1-yl]-4,4,5,5-tetrafluoro-3-oxocyclopent-1-en-1-olate (15-betaine):

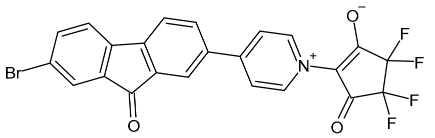

Compound **15** (101 mg, 0.3 mmol, 1.0 eq) and DMSO (4 mL) were placed into 12 mL "pressure tube". The solution was cooled to 0 °C, and octafluorocyclopentene (**4**) (510 mg, 320 µL, 2.4 mmol, 8 eq) was added, as described in **GP5**. The tube was stopped by a screwcap, and the reaction mixture stirred at 60 °C for 68 h. The reaction mixture was diluted with dioxane (4 mL); 1 mL of saturated aqueous $NaHCO_3$ added, and the entire mixture stirred for 45 min to give a precipitate consisting of inorganic (higher density) and organic (lower density) compounds. The suspension was diluted with acetone, shaken and, after the inorganic salts were settled dawn, the supernant layer (containing suspended organic material with low solubility and lower density) was decanted. This operation was repeated several times and collected supernant layers was filtered off washed with acetone. Acetone was removed at low vacuum (1 mbar) and the residual solid was freeze-dried to give target compound **15**-betaine (134 mg, 90%).

^{1}H NMR (500MHz, DMSO-d_6): δ = 9.32 (dm, J=7.4 Hz, 2H), 8.69 (dm, J=7.4 Hz, 2H), 8.41 (dd, J=8.0, 1.8 Hz, 1H), 8.35 (dd, J=1.8, 0.5 Hz, 1H), 8.15 (dd, J=8.0, 0.5 Hz, 1H), 7.96 (dd, J=8.0, 0.6 Hz, 1H), 7.93 (dd, J=8.0, 1.8 Hz, 1H), 7.86 (dd, J=1.8, 0.6 Hz, 1H) ppm. ^{13}C NMR (126MHz, DMSO-d_6): δ = 190.8, 152.4, 146.2, 142.1, 141.8, 138.0, 135.72, 135.65, 134.9, 134.1, 127.1, 124.3, 124.0, 123.7, 123.6, 122.7 ppm, due to C-F coupling the ^{13}C signals of the betaine fragment were not resolved. ^{19}F NMR (376MHz, DMSO-d_6): δ = -126.04 ppm (s, 4F). HRMS (ESI) calcd for $C_{23}H_{10}BrF_4NO_3Na$ $[M+Na]^+$ 525.9672 found 525.9683. HRMS (ESI) calcd for $C_{24}H_{14}BrF_4NO_4Na$ $[M+MeOH+Na]^+$ 557.9935 found 557.9946.
